# Adjuvant-free peptide vaccine targeting Clec9a on dendritic cells can induce robust antitumor immune response through Syk/IL-21 axis

**DOI:** 10.7150/thno.56406

**Published:** 2021-05-24

**Authors:** Shanshan Gou, Shuai Wang, Wenwen Liu, Guanyu Chen, Dongyang Zhang, Jiangfeng Du, Zhongyi Yan, Hongfei Wang, Wenjie Zhai, Xinghua Sui, Yahong Wu, Yuanming Qi, Yanfeng Gao

**Affiliations:** 1 School of Life Sciences, Zhengzhou University, Zhengzhou 450001, China.; 2 School of Pharmaceutical Sciences (Shenzhen), Sun Yat-sen University, Shenzhen 518107, China.

**Keywords:** Clec9a, dendritic cell, peptide vaccine, cancer immunotherapy, IL-21

## Abstract

Dendritic cells (DCs) can process the antigens of cancer vaccine and thus stimulate the CD8^+^ T cells to recognize and kill the tumor cells that express these antigens. However, lack of promising carriers for presenting the antigens to DCs is one of the main barriers to the development of clinically effective cancer vaccines. Another limitation is the risk of inflammatory side effects induced by the adjuvants. It is still unclear how we can develop ideal adjuvant-free DC vaccine carriers without adjuvants.

**Methods:** A 12-mer peptide carrier (CBP-12) with high affinity for Clec9a expressed on DCs was developed using an *in silico* rational optimization method. The therapeutic effects of the adjuvant-free vaccine comprising CBP-12 and exogenous or endogenous antigenic peptides were investigated in terms of antigen cross-presentation efficacy, specific cytotoxic T lymphocyte response, and antitumor activity. We also explored the mechanism involved in the antitumor effects of the adjuvant-free CBP-12 vaccine. Finally, we assessed the effects of the CBP-12 conjugated peptide vaccine combined with radiotherapy.

**Results:** Here, we developed CBP-12 as a vaccine carrier that enhanced the uptake and cross-presentation of the antigens, thus inducing strong CD8^+^ T cell responses and antitumor effects in both anti-PD-1-responsive (B16-OVA) and -resistant (B16) models, even in adjuvant-free conditions. CBP-12 bound to and activated Clec9a, thereby stimulating Clec9a^+^ DC to product IL-21, but not IL-12 by activating of Syk. The antitumor effects of the CBP-12 conjugated peptide vaccines could be blocked by an IL-21 neutralizing antibody. We also observed the synergistic antitumor effects of the CBP-12 conjugated peptide vaccine combined with radiotherapy.

**Conclusions:** CBP-12 could serve as an adjuvant-free peptide vaccine carrier for cancer immunotherapy.

## Introduction

In recent years, immunotherapy has emerged as an important mode of cancer therapy, independent of conventional treatments 1, 2. A number of immunotherapeutic drugs have been approved for treating malignancies 3, 4. For instance, immune checkpoint inhibitors (ICIs) are now available for the treatment of cancers that include advanced melanoma, non-small cell lung cancer, and merkel cell carcinoma 5. However, only a small fraction of patients benefits from ICIs. There are other promising strategies, such as therapeutic vaccines based on tumor antigens, including neoantigens or tumor-associated antigens (TAAs), that stimulate T cell responses and elicit synergistic effects with ICIs 6, 7.* In situ* cancer vaccination displayed clinical benefits for the treatment of advanced lymphoma, and a combination of vaccination with anti-PD-1 therapies achieved tumor regression 8. It was reported that these therapeutic vaccines could successfully heat up so-called “cold tumors” with a relatively low level of immune cell infiltration 9. Therefore, combination with these vaccines could improve the overall response to immunotherapy in patients who do not benefit from ICIs 10.

Cross-presentation by antigen-presenting cells, especially DCs, is the key step by which cancer vaccines stimulate tumor-specific T lymphocytes 11. The mouse CD8α^+^ DC subset excels at antigen cross-presentation, and thereby stimulates robust cytotoxic T lymphocyte (CTL) responses. Clec9a, also known as DNGR-1, is a C-type lectin receptor expressed on mouse CD8α^+^ DCs and plasmacytoid DCs (pDCs) 12, 13, and is also selectively expressed on human BDCA3^+^ DCs 14, 15. Clec9a specifically recognizes actin filaments (F-actin) exposed on necrotic cells, leading to the subsequent routing of necrotic cells into the cross-priming pathway 16, 17. Endocytic receptors that are expressed on CD8α^+^ DCs, such as Clec9a 13, DEC-205, and Clec12a, can mediate antigen internalization 13, 18. Therefore, these receptors could be utilized as cancer vaccine delivery targets. It was reported that in the presence of an adjuvant, antigens targeting DEC-205 and Clec9a, but not Clec12a, induced the production of CTLs, with the greatest response elicited by targeting Clec9a 19. Further studies showed that antigens delivered via a Clec9a antibody, in the presence of an adjuvant, enhanced vaccine efficacy to induce CTLs, type I interferon (IFN) production, antibody production, and antitumor immunity 12, 13.

Although this antibody-based Clec9a delivery system with an adjuvant is a promising strategy for tumor vaccination, there are two concerns. First, it is difficult to conjugate or fuse the antibodies used for antigen delivery with the antigens 20. Second, adjuvants can randomly activate innate immune cells and induce inflammatory side effects 21, 22. To overcome these problems, we sought to develop small peptide antigen carriers that can effectively target Clec9a expressed on DCs. We also investigated whether these peptide carriers conjugated to cancer vaccines are effective in the absence of an adjuvant.

In this study, we developed a 12-mer Clec9a binding peptide (CBP-12) using a computer-aided *in silico* method 23. The high affinity CBP-12 enhanced antigen uptake and cross-presentation by Clec9a^+^ DCs to stimulate antigen-specific CD8^+^ T cell responses. The CBP-12 conjugated peptide vaccines significantly inhibited tumor growth, in the absence of an adjuvant, in anti-PD-1-responsive and -resistant tumor models. In terms of the underlying mechanisms, we found that CBP-12 stimulated Clec9a^+^ DCs to release IL-21 through the activation of Syk. Finally, we observed synergistic effects of the CBP-12 conjugated peptide vaccine in combination with radiotherapy.

## Methods

### Mice

Female C57BL/6 mice, aged 6-8 weeks, were purchased from Vital River Laboratory Animal Technology Co. Ltd (Beijing, China). Clec9a^-/-^ mice were purchased from The Jackson Laboratory (Bar Harbor, ME, USA). OT-1 mice (OVA_257-264_ TCR transgenic mice) were kindly provided by Prof. Xuanming Yang (School of Life Science and Technology, Shanghai Jiao Tong University, China). All mice were maintained in a specific pathogen-free facility. Animal welfare and experimental procedures were carried out following the national and institutional guidelines for animal care, and approved by the Ethics Committee of Sun Yat-sen University (SYSU-IACUC-2019-B720).

### Cells

B16-OVA and B16 cells were cultured in RPMI 1640 (Biological Industries, Kibbutz Beit Haemek, lsrael). Human papilloma virus (HPV) E6/E7 antigen transgenic cell line TC-1 (murine lung cancer) was maintained in Prof. Hua Peng's laboratory (Institute of Biophysics, Chinese Academy of Sciences, China). TC-1 and HEK-293T cells were cultured in DMEM (Biological Industries, Kibbutz Beit Haemek, lsrael). Mouse FL-DCs were generated by culturing bone marrow (BM) cells in the presence of 200 ng/mL of Flt3L (eBioscience, San Diego, CA, USA) for 10-12 days with semi-medium changing every 4 days. CD8^+^ T cells of OT-1 mice were isolated from lymph nodes by EasySep™ Mouse CD8^+^ T Cell Isolation Kit (STEMCELL, Vancouver, BC, Canada). All the culture medium was supplemented with 10% fetal bovine serum (Biological Industries, Kibbutz Beit Haemek, lsrael), penicillin (Solarbio, Beijing, China), streptomycin (Solarbio, Beijing, China) and 2-macraptoethanol (Life technology, Carlsbad, CA, USA), and cells were cultured at 37 °C under humidified air with 5% CO_2_.

### Antibodies

Streptavidin-PE (SA-PE), anti-mouse-CD45-FITC (30-F11), anti-mouse CD11c-PerCP-efluor 450 (N418), anti-mouse CD11b-APC (M1/70), anti-mouse-CD3-PerCP-eFlour710 (17A2), anti-mouse-CD8-APC (53-6.7), Rat IgG2a-APC kappa Isotype Control (eBR2a), anti-mouse-CD3-FITC (17A2), anti-mouse-CD8-PE (53-6.7), anti-mouse-IFN-γ-APC (XMG1.2), anti-mouse-Perforin-APC (eBioMAK-D), anti-mouse-Granzyme B-PerCP-eFlour710 (NGZB), Rat IgG1-APC kappa Isotype Control (eBRG1), Rat IgG2a-PerCP-eFlour710 kappa Isotype Control (eBR2a), anti-mouse-OVA_257-264_ (SIINFEKL/H-2K^b^)-PE (eBio25-D1.16), Mouse IgG1-PE kappa Isotype Control (P3.6.2.8.1), anti-mouse-IL-21-PE (FFA21), Rat IgG2a-PE kappa Isotype Control (eBR2a), anti-mouse-Clec9a-PE (42D2), anti-mouse CD11c-APC (N418) and Rat IgG1-PE kappa Isotype Control (eBRG1) were purchased from eBioscience (San Diego, CA, USA). The anti-mouse/rat XCR1-Brilliant Violet 650^TM^ (ZET) and anti-mouse/rat XCR1-APC (ZET) were purchased from Biolegend (San Diego, CA, USA). The anti-mouse IL-21 (FFA21) neutralized antibody was purchased from Thermo Fisher Scientific (Waltham, MA, USA). The phospho-Syk (Tyr323) antibody was purchased from CST (Boston, USA). The Anti-Histone H3 antibody was purchased from Abcam (Cambridge, United Kingdom).

### Peptide synthesis

Peptides were synthesized according to the standard Fmoc solid phase strategy and purified by reverse phase-high performance liquid chromatography (RP-HPLC), and the products with purity of more than 95% were obtained. The molecular weights were assessed by mass spectrometry (MS). For binding assay, GGGK^biotin^ was linked to the peptide at carboxyl terminal. For* in vivo* tests in mice, OVA_257-264_ was added to the peptide by a GGG linker at carboxyl terminal. Peptide sequences were as follows: GAGAAGGAGGGG-GGG-K^biotin^ (GA^biotin^); WPRFHSSVFHTH-GGG-K^biotin^ (WH^biotin^); WPRFHSSVRHTH-GGG-K^biotin^ (CBP-12^biotin^); SIINFEKL (OVA_257-264_); GAGAAGGAGGGG-GGG-SIINFEKL (GA-OVA); WPRFHSSVRHTH-GGG-SIINFEKL (CBP-12-OVA); EGSRNQDWL (gp100_25-33_); WPRFHSSVRHTH-GGG-EGSRNQDWL (CBP-12-gp100); RAHYNIVTF (E7_49-57_); WPRFHSSVRHTH-GGG-RAHYNIVTF (CBP-12-E7). GA peptide was used as a negative control peptide 24.

### Peptide binding assay by flow cytometry

HEK-293T cells were transiently transfected with pIRES2-EGFP-mClec9a plasmid, or the mock pIRES2-EGFP plasmid for binding assay. Cells (3 × 10^5^) were incubated with rat serum to block Fc receptors and different biotinylated peptides for 30 min on ice. After washing three times, cells were stained with streptavidin (SA)-PE for 30 min on ice, and the samples were analyzed for binding affinity by a flow cytometry after washing once.

### Binding affinity measurement by MST

The affinity of the peptide to mouse Clec9a (mClec9a) protein was detected by microscale thermophoresis (MST) (NanoTemper Technologies GmhH, Germany). Briefly, mClec9a protein (Sino Biological, Beijing, China) was labeled with Red-NHS647 dye (NanoTemper Technologies GmhH, Germany), and the successfully labeled protein was used for affinity measurements. Peptides were diluted into serial concentrations with MST buffer. Equal volume of dye-labeled mClec9a protein was incubated with peptides at room temperature, the mixture was loaded onto capillaries (NanoTemper Technologies GmhH, Germany) and immediately analyzed by an MST instrument. The data were estimated by MO. Affinity Analysis v2.2.4.

### Cellular uptake

Mouse splenocytes (5 × 10^5^) were incubated with biotinylated GA peptide and CBP-12 for 30 min on ice, and then cells were washed three times and cultured for 2 h at 37 °C. Spleen cells were stained with anti-mouse CD45-FITC (30-F11), anti-mouse CD11c-PerCP-efluor 450 (N418), anti-mouse XCR1-BV650 (ZET) and anti-mouse CD11b-APC (M1/70). Then cells were fixed, permeabilized and further stained with SA-PE for peptide uptake analysis.

Biotinylated peptide was mixed with SA-PE in a molar ratio of 1: 3 to obtain SA-PE labeled peptide (peptide^biotin-SA-PE^). FL-DCs from wild type (WT) or Clec9a^-/-^ mice was harvested and washed twice with PBS before use. In some cases, cells were pre-incubated with 10 μg/mL of chlorpromazine 25 (MedChem Express, Monmouth Junction, NJ, USA), or 50 μg/mL of genistein 25 (MedChem Express, Monmouth Junction, NJ, USA) for 1 h, and then incubated with 50 μM GA^biotin-SA-PE^ or CBP-12^biotin-SA-PE^ for 2 h at 37 °C. After washing, cells were incubated with Hoechst 33342 (Beyotime Biotechnology, Beijing, China) for 1 h to stain the nucleus. Cells were analyzed with a confocal laser scanning microscopy (Leica TCS SP8, Wetzlar, Germany).

### *In vitro* cross-presentation of OVA_257-264_ antigen and T cell priming by FL-DCs

*In vitro* stimulation T cell assay by OVA_257-264_, GA-OVA, CBP-12-OVA or PBS was performed as described previously 23. FL-DCs were incubated with 10 μg/mL OVA_257-264_, GA-OVA, CBP-12-OVA or PBS for 1.5 h at 4 °C, and then the peptide-loaded FL-DCs were washed three times. Meanwhile, CD8^+^ T cells were isolated using EasySep^TM^ CD8^+^ T cell negative selection kit (STEMCELL, Vancouver, BC, Canada) from OT-1 mice. After labeling CD8^+^ T cells with 2 μΜ CFSE (eBioscience, San Diego, CA, US), 1 × 10^5^ CD8^+^ T cells per well were plated into round-bottom 96-well plates. After 30 min, 1 × 10^4^ peptide-loaded FL-DCs per well were gently added to the plates and incubated with CD8^+^ T cells for 72 h. Proliferation of DC-primed CD8^+^ T cells was determined by CFSE dilution. The cells were incubated with anti-mouse-CD3-PerCP-eFlour710 (17A2) and anti-mouse-CD8-APC (53-6.7) or Rat IgG2a-APC kappa Isotype Control (eBR2a), CD8^+^ T cells proliferation were detected by a flow cytometry. Supernatant was assayed for IFN-γ by a Mouse IFN-gamma ELISA kit (Thermo Fisher Scientific, Waltham, MA, USA).

### Tumor bearing experiments

Female C57BL/6 mice were subcutaneously (s.c.) injected on the right back with 2 × 10^5^ B16-OVA or B16 cells. On day 3 of the tumor cell inoculation, mice were randomly allocated into groups and vaccinated with 20 μg peptides or normal saline weekly for total of three times. Tumor volume was measured every other day. After five days of last immunization, mice were sacrificed. For IL-21 blocking experiment *in vivo*, mice were intraperitoneally (i.p.) injected 100 μg IL-21 neutralized antibody (FFA21) every eight days for total of twice.

Tumor tissue cell suspensions were stained with anti-mouse-CD45-FITC (30-F11), anti-mouse-CD3-PerCP-eFlour710 (17A2) and anti-mouse-CD8-APC (53-6.7) or Rat IgG2a-APC kappa Isotype Control (eBR2a) for tumor infiltrating CD8^+^ T cell analysis.

For intracellular cytokine staining (ICS), single cell suspension of splenocytes and draining lymph node (dLN) was stimulated with 10 μg/mL OVA_257-264_ peptide in the presence of protein transport inhibitor cocktail (eBioscience, San Diego, CA, US) for 6 h. Cells were then stained with surface markers CD3 and CD8 prior to fixation and permeabilization. Permeability cells were then stained with intracellular anti-mouse-IFN-γ-APC (XMG1.2), anti-mouse-Perforin-APC (eBioMAK-D), anti-mouse-Granzyme B-PerCP-eFlour710 (NGZB) antibody or isotype control antibody. Cells were collected and suspended in PBS containing 0.5% fetal bovine serum, incubated with the corresponding antibodies for 30 min on ice, washed once with PBS and analyzed by a FACS Calibur (Becton Dickinson, Franklin Lake, NJ, USA) flow cytometry.

### *In vitro* neutralizing IL-21 inhibited cross-presentation of antigen and T cell priming by DC

FL-DCs were incubated with 6 μM OVA_257-264_, GA-OVA, CBP-12-OVA or PBS in the presence or absence of 5 μg/mL anti-mouse IL-21 (FFA21) for 2 h at 37 °C, and then washed twice with serum-free medium. Cells stained with anti-mouse-CD11c-APC (N418) and anti-mouse-OVA_257-264_ (SIINFEKL/H-2K^b^)-PE (eBio25-D1.16) or anti-mouse IgG1-PE kappa Isotype Control (P3.6.2.8.1) for OVA_257-264_/H-2K^b^ complex percentage analysis.

As mentioned before, peptide-loaded FL-DCs were co-incubated with CD8^+^ T cell in the presence or absence of 5 μg/mL anti-mouse IL-21 (FFA21) for 72 h at 37 °C. Proliferation of DC-primed CD8^+^ T cells was determined by CFSE dilution. Cells were incubated with anti-mouse-CD3-PerCP-eFlour710 (17A2) and anti-mouse-CD8-APC (53-6.7) or Rat IgG2a-APC kappa Isotype Control (eBR2a), CD8^+^ T cells proliferation was detected by a flow cytometry. The IFN-γ content was measured by a Mouse IFN-gamma ELISA kit (Thermo Fisher Scientific, Waltham, MA, USA).

### Intracellular cytokine staining for IL-21

FL-DCs were incubated with 100 μg/mL OVA_257-264_, GA-OVA, CBP-12-OVA or PBS and protein transport inhibitor cocktail at 37 °C for 6 h. For Syk phosphorylation inhibition assay, FL-DCs were incubated with 2.5 μM R406 (MCE, New Jersey, USA) for 1 h and then cultured with 100 μg/mL CBP-12-OVA and protein transport inhibitor cocktail at 37 °C for 6 h. IL-21 positive frequency was detected by ICS. R406 is an ATP-competitive Syk kinase inhibitor 26. Cells stained with anti-mouse-CD11c-APC (N418), anti-mouse/rat XCR1-Brilliant Violet 650™ (ZET) and anti-mouse-IL-21-PE (FFA21) or Rat IgG2a-PE kappa Isotype Control (eBR2a) for IL-21 production were analyzed by a flow cytometry.

### IL-21 ELISA assay

FL-DCs were labeled with anti-mouse/rat XCR1-APC antibody (ZET), and the APC positive cells were purified by a Mouse APC Positive Selection Kit (STEMCELL, Vancouver, BC, Canada). cDC1s were incubated with 100 μg/mL peptides for 48 h at 37 °C. The supernatant was harvested for detecting IL-21 production determent by using an ELISA kit (MULTI SCIENCES, Hangzhou, China).

### Western blot

FL-DCs were incubated with 100 μg/mL OVA_257-264_, GA-OVA, CBP-12-OVA, PBS and 2 mM sodium orthovanadate (pH 10.0) for 30 min at 37 °C. FL-DCs were lysed by using the total protein extraction buffer (Tris 7.58 g/L, SDS 20 g/L, β-mercaptoethanol 20 mL/L, Glycerol 100 mL/L, Bromophenol Blue 0.2 g/L, pH6.8), and the lysates were denatured immediately at 100 °C for 10 min. Subsequently, a fixed amount of protein extracts was subjected to separation by SDS-PAGE and subsequent visualization by western blot with corresponding antibodies indicated.

### Combination therapy with radiotherapy

C57BL/6 mice were s.c. injected with 2 × 10^5^ B16-OVA cells into the right flank. When tumors reached 40-70 mm^3^, the mice were grouped randomly, and some mice received 15 Gy of local irradiation (IR) twice on day 8 and 9. The next day, mice were vaccinated with 20 μg CBP-12-OVA or normal saline (negative control) every four days for total of three times. Tumor cells were stained with anti-mouse-CD45-FITC (30-F11), anti-mouse-CD11c-APC (N418), anti-mouse-Clec9a-PE (42D2) or Rat IgG1-PE Isotype Control (eBRG1) for CD11c^+^Clec9a^+^ cell analysis.

For TC-1 tumor model, mice were injected s.c. with 1.5 × 10^5^ TC-1 cells into the right flank. When the tumors reached 200 mm^3^, mice received 20 Gy of local IR, and 20 μg peptide was immunized weekly for total of three times.

### Statistical analysis

Statistical analysis was conducted with a single-tailed Student's *t* test for the differences between groups. The tumor growth curves were performed using two-way ANOVA. Kaplan-Meier curves and the log-rank test were used for analyzing overall survival rate of the mice. The data were shown as the mean ± SEM unless otherwise stated. Statistical significance was defined as *P < 0.05, **P < 0.01, ***P < 0.001.

## Results

### CBP-12 shows high affinity for Clec9a through which it enhances OVA_257-264_ cross-presentation

**Figure 1:**
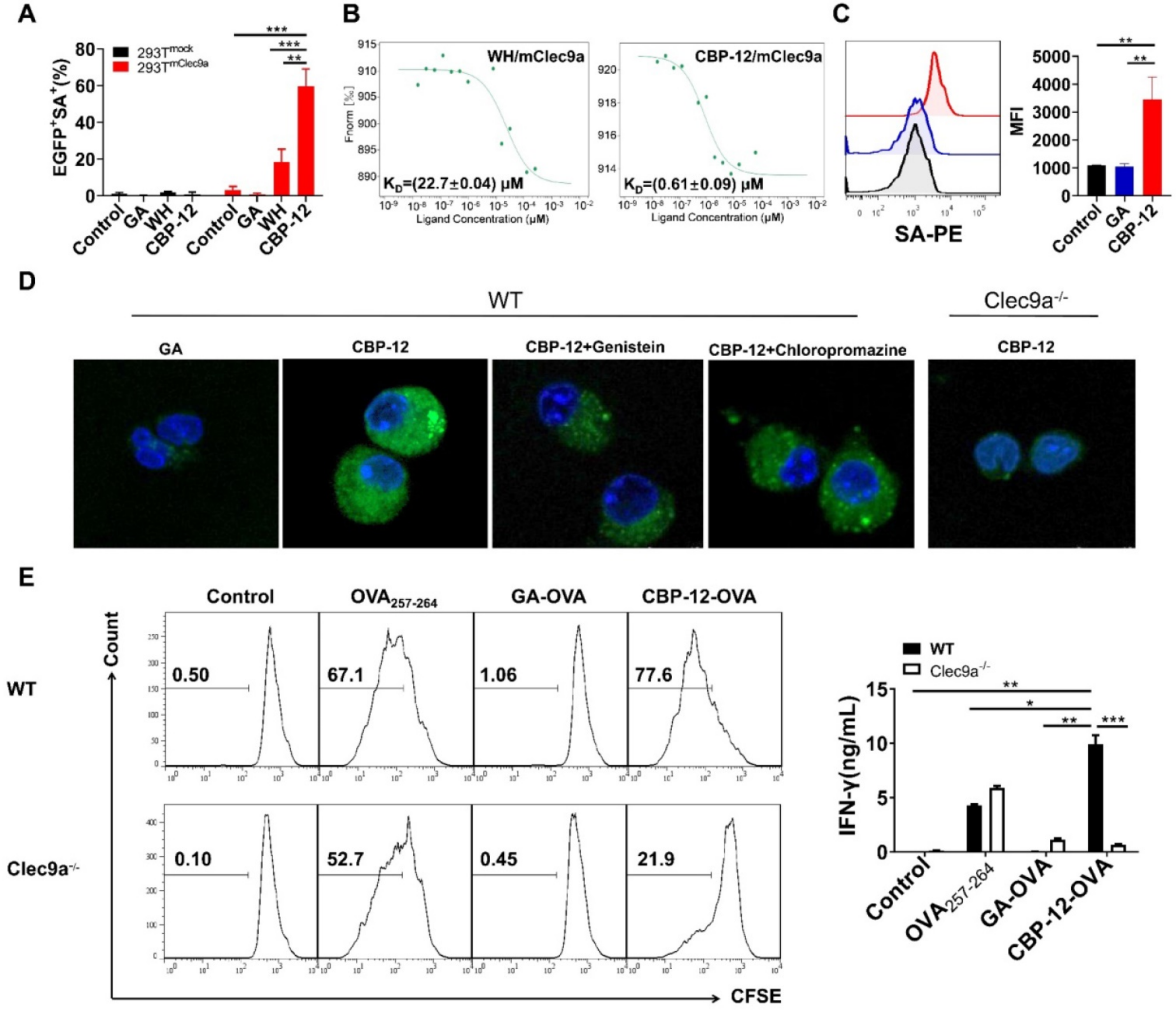
**Effects of CBP-12 on OVA_257-264_ antigen presentation by FL-DCs (Flt3L induced DCs) *in vitro*.** PBS was used as a negative control. (A) Binding of CBP-12 and WH peptide to HEK-293T^mock^ and HEK-293T^mClec9a^ cells. GA was used as a negative control peptide. Biotinylated peptides (100 µM) were incubated with the transfected HEK-293T cells for 30 min on ice, washed three times with FCS buffer containing 2 mM EDTA, and stained with SA-PE to label the biotinylated peptides bound to the transfected cells. (B) The K_D_ values of the CBP-12 and WH peptide to mClec9a protein tested using the MST. (C) Uptake of biotinylated peptide by spleen cDC1s (CD45^+^CD11c^+^XCR1^+^) *in vitro*. Mouse splenocytes were incubated with 6.25 µM of biotinylated GA and CBP-12 for 30 min on ice, washed three times, and then cultured for 2 h at 37 °C. The cells were fixed, permeabilized, and stained with SA-PE for peptide uptake analysis. (D) Targeting Clec9a enhances the CBP-12 uptake by FL-DCs via endocytosis. FL-DCs from WT and Clec9a^-/-^ mice were incubated with 50 µM peptide^biotin-SA-PE^ in the presence or absence of the indicated inhibitors for 2 h. Cell nuclei were stained with Hoechst 33342 (blue). The FL-DCs that internalized peptide^biotin-SA-PE^ are labeled green. Scale bar, 10 µm. (E) FL-DCs of WT and Clec9a^-/-^ mice stimulated naïve T cell proliferation and IFN-γ secretion was measured by an ELISA. FL-DCs were incubated with the indicated peptides or normal saline for 1.5 h at 4 °C, after which the FL-DCs were harvested. CD8^+^ T cells were isolated from the lymph nodes of OT-1 mice and labeled with 2 µM CFSE. Then, 1 × 10^5^ CFSE-labeled CD8^+^ T cells were incubated with 1 × 10^4^ peptides loaded FL-DCs for 72 h at 37 °C. Data are presented as the mean ± SEM. *P < 0.05, **P < 0.01, ***P < 0.001. Data are representative of three independent experiments.

To obtain peptides with high affinity and selectivity toward mClec9a, *in silico* mutagenesis was applied using the WH/mClec9a model, as previously described 23. We identified 15 optimized peptides that theoretically displayed high binding affinity to mClec9a. The binding assay showed that CBP-12 exhibited higher binding affinity than the other peptides in mClec9a-overexpressing HEK-293T cells (Figure 1A, S1, and S2). CBP-12 did not bind to the mock plasmid transfected HEK-293T cells (Figure 1A and S1), indicating that CBP-12 specifically binds to mClec9a. As shown in Figure 1B, the K_D_ values of CBP-12 and WH peptide for mClec9a were 0.61 ± 0.09 μM and 22.7 ± 0.04 μM, respectively. Thus, the affinity of CBP-12 for mClec9a was 37.2-fold higher than that of WH peptide. Moreover, CBP-12 exhibited strong uptake by spleen cDC1s both *in vitro* and *in vivo*, and it was effectively taken up by cDC1s within 15 min *in vivo* (Figure 1C and S3). CBP-12 was stable in mouse serum for 1 h (Figure S4), which might facilitate its activity *in vivo*. As shown in Figure 1D, incubating Flt3L-induced DCs (FL-DCs) with CBP-12 resulted in a strong fluorescence signal. By contrast, the fluorescence signal was much lower in FL-DCs derived from Clec9a^-/-^ mice. This uptake of CBP-12 was inhibited by amiloride or chlorpromazine (inhibitors of clathrin-mediated endocytosis), which suggests that multiple endocytotic pathways are involved in the internalization of CBP-12 by Clec9a^+^ DCs. Moreover, flow cytometry analysis confirmed the uptake of CBP-12 by cDC1s (Figure S5). To further investigate whether CBP-12 targeting Clec9a resulted in efficient antigen presentation for T cell cross-priming, FL-DCs loaded with peptides were incubated with CD8^+^ T cells (OVA_257-264_ specific cells, from OT-1 mice) for 72 h. CBP-12-OVA was more effective in stimulating the proliferation of antigen-specific CD8^+^ T cells and IFN-γ secretion compared with OVA_257-264_ and GA-OVA. Interestingly, CD8^+^ T cells could not be activated by FL-DCs derived from the Clec9a^-/-^ mice (Figure 1E). In addition, CBP-12-OVA stimulated the proliferation of CD8^+^ T cells, at concentrations ranging from 1.25 to 10 μg/mL, which was stronger than WH-OVA peptide (Figure S6). These results indicate that CBP-12 has great potential to selectively enhance the antigen cross-presentation capability of Clec9a^+^ FL-DCs and promote T cell activation.

### Antigens conjugated to CBP-12 elicited antitumor immune responses in the absence of an adjuvant

In preliminary experiments, we compared the effects of incomplete Freund's adjuvant, polyI:C, and CpG to induce CTL responses *in vivo*. We found that co-immunization of mice with CpG and the peptide vaccine induced the greatest CTL response (data not shown). Therefore, we first used CpG as the adjuvant to test the immunization activity of the CBP-12 conjugated peptide vaccine. In the B16-OVA tumor model, immunization with CBP-12-OVA in the presence of CpG inhibited B16-OVA tumor growth and enhanced antigen-specific CD8^+^ T cells responses (Figure S7). The antitumor effect of the CBP-12-OVA conjugated peptide vaccine was significantly impaired in Clec9a^-/-^ mice (Figure S7E), indicating that CBP-12 specifically targets Clec9a *in vivo* and the antitumor effects of CBP-12-OVA are dependent on Clec9a. Surprisingly, in naïve mice, in the absence of an adjuvant, CBP-12-OVA induced antigen-specific CTL responses (Figure S8)*.*

**Figure 2:**
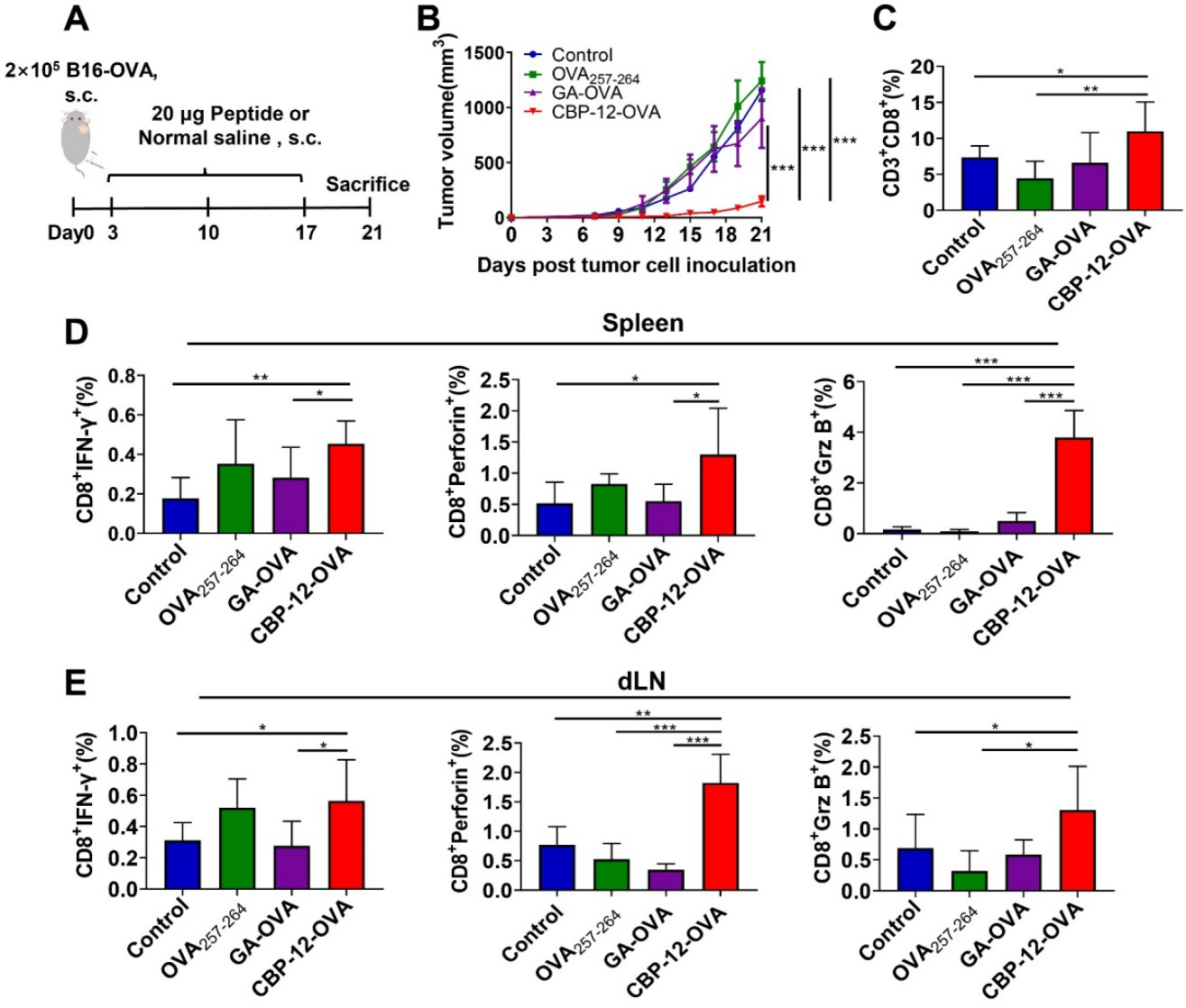
** Antitumor effects of CBP-12-OVA in the B16-OVA melanoma model.** (A) Model schema. B16-OVA cells (2 × 10^5^) were injected s.c. into the right flank of C57BL/6 mice. Starting on day 3, the mice were vaccinated with 20 µg of the indicated peptides or with normal saline (negative control), a total of three times at 1-week intervals. Five days after the last dose, the mice were sacrificed. (B) Tumor volume. (C) Percentage of tumor-infiltrating CD8^+^ T cells. (D, E) Activated cytotoxic CD8^+^ T cells in the spleen and dLN were counted by flow cytometry. Splenocytes and dLN cells were restimulated with 10 µg/mL OVA_257-264_ peptide for 6 h* in vitro,* and ICS was performed to determine the antigen-specific T cell responses. The tumor growth curves were compared using two-way ANOVA. Data are presented as the mean ± SEM. *P < 0.05, **P < 0.01, ***P < 0.001. *n* = 5 per group.

To determine whether adjuvant-free CBP-12-OVA elicits antitumor immune responses, we established a B16-OVA melanoma model (Figure 2A). The parent peptide WH conjugated peptide vaccine (WH-OVA) did not inhibit tumor growth in the absence of an adjuvant (Figure S9). Tumor volume and tumor weight were significantly reduced in mice treated with adjuvant-free CBP-12-OVA as compared with the normal saline, OVA_257-264_, and GA-OVA groups (Figure 2B and S10). There were no obvious differences in the weights of the dLN and spleen among the groups (Figure S10). Among eight mice in each group, three, three, two, and eight were still alive at the end of treatment in the normal saline, OVA_257-264_, GA-OVA, and CBP-12-OVA groups, respectively. Although there were no tumor-free mice, the frequency of memory T cells was significantly greater in the CBP-12-OVA group than in the control groups (Figure S11). These results indicate that the CBP-12-OVA peptide vaccine induced antitumor immune responses and memory T cells.

To verify whether CBP-12-OVA directly inhibits cancer cell proliferation *in vitro*, we determined the viability of B16-OVA cells using an MTT assay after incubating the cells with different peptides for 24, 48, or 72 h. CBP-12-OVA did not exert obvious cytotoxic effects at concentrations ranging from 12.5 to 100 μg/mL (Figure S12). Furthermore, the proportion of tumor-infiltrating CD8^+^ T cells in the CBP-12-OVA immunized group was greater than that in the other groups (Figure 2C and S13A). The infiltration of CD4^+^ T cells and NK cells in the tumor was also detected. As shown in Figure S14A, CBP-12-OVA significantly increased the proportion of tumor-infiltrating CD4^+^ T cells but not NK cells. These results suggest that CD4^+^ T cells might participate in the antitumor effects of the CBP-12-OVA peptide vaccine. We also determined the tumor infiltration of the immunosuppressive cells MDSCs, Th2 cells, and Treg cells by flow cytometry. Interestingly, the proportion of tumor-infiltrating Treg cells was significantly reduced in the CBP-12-OVA peptide vaccine treatment group (Figure S14B), but MDSCs and Th2 cells were not. These results indicate that CBP-12-OVA inhibited tumor growth by increasing the number of tumor-infiltrating CD8^+^ and CD4^+^ T lymphocytes and reducing Treg cells. The spleen and dLN from mice treated with CBP-12-OVA and re-stimulated with OVA_257-264_ *in vitro* generated higher levels of IFN-γ, perforin, and Granzyme B (Grz B) than that in the other groups (Figure 2D, E and S13B, C), which demonstrates the induction of an antigen-specific CTL response.

To investigate whether the peptide was contaminated with an endotoxin, which might act as adjuvant, we examined the traces of endotoxin in CBP-12-OVA but detected no endotoxin contamination (Figure S15A). It was reported that endotoxins upregulate the gene expression of TNF-α and IL-6 in RAW264.7 cells 27. We found that CBP-12-OVA did not upregulate the mRNA expression levels of TNF-α or IL-6 (Figure S15B, C), suggesting that CBP-12-OVA was not contaminated with endotoxin. Overall, these results indicate that CBP-12 promoted the immunization activity of the antigenic peptide to increase tumor-infiltrating T lymphocytes and induce antigen-specific CD8^+^ T cell responses in the spleen and dLN targeting B16-OVA tumors, without requiring an adjuvant.

### Endogenous antigens conjugated to CBP-12 prevent B16 melanoma growth in the absence of an adjuvant

**Figure 3:**
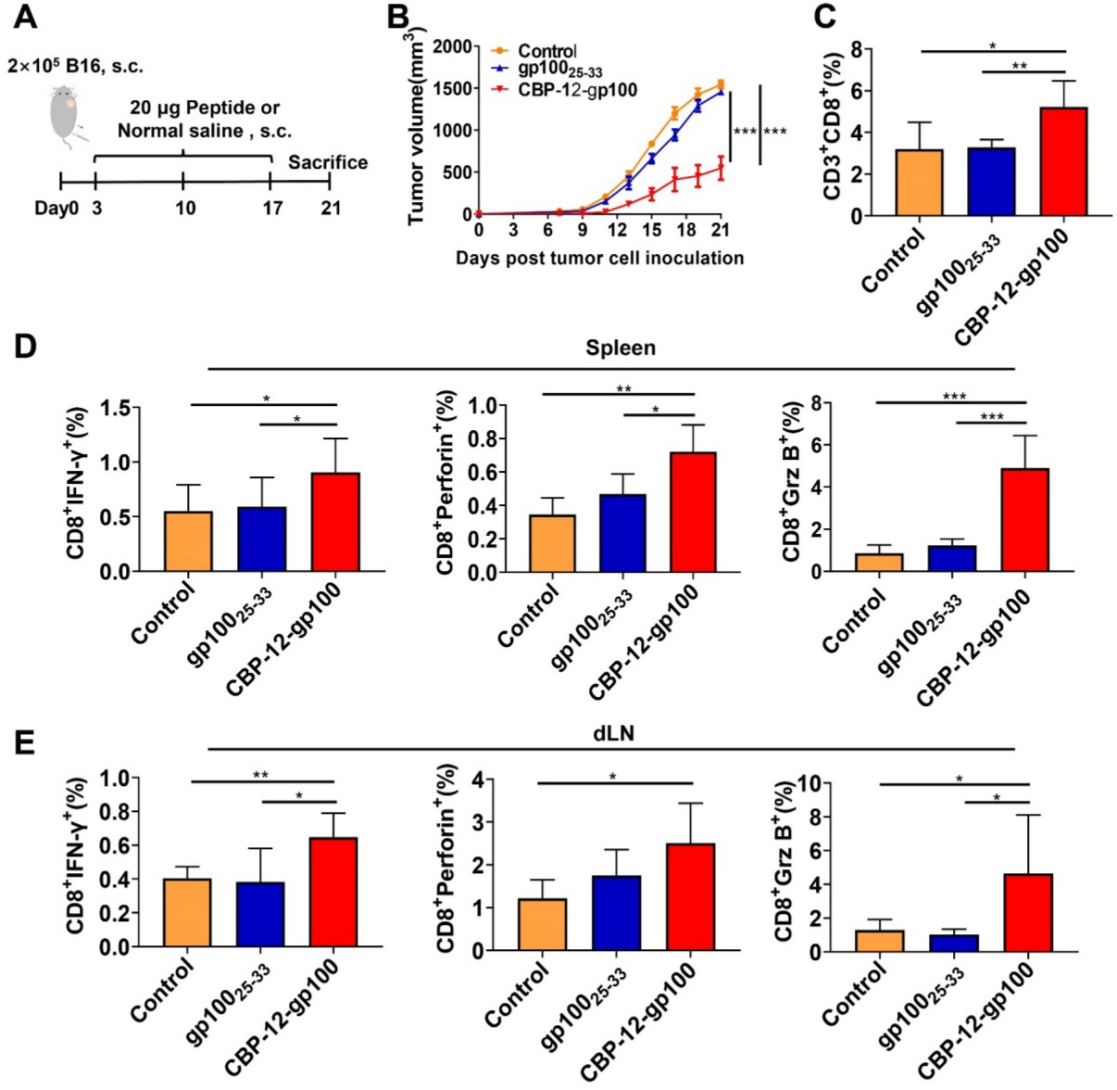
** Immunotherapy of B16 melanoma by targeting tumor endogenous antigens via Clec9a.** (A) Model schema. B16 melanoma cells (2 × 10^5^) were s.c. injected into C57BL/6 mice. Starting on day 3, the mice were vaccinated with 20 µg of the indicated peptides or with normal saline (negative control), a total of three times at 1-week intervals. Tumor volume was measured every 2 days. Five days after the last dose, the mice were sacrificed. (B) Tumor volume. (C) Percentage of tumor-infiltrating CD8^+^ T cells. (D, E) Activated cytotoxic CD8^+^ T cells in the spleen and dLN were counted by flow cytometry. The splenocytes and dLN cells were restimulated with 10 µg/mL OVA_257-264_ peptide for 6 h *in vitro,* and ICS was performed to determine the antigen-specific T cell responses. The tumor growth curves were compared using two-way ANOVA. Data are presented as the mean ± SEM. *P < 0.05, **P < 0.01, *** *P*< 0.001. *n* = 5 per group.

To further determine whether the endogenous antigenic peptides delivered by CBP-12 also induce immune responses in the anti-PD-1-resistant tumor B16 model, we synthesized a CBP-12-gp100 conjugated peptide vaccine (Figure 3A), in which the antigenic peptide (glycoprotein 100) was selected from melanocyte lineage-specific endogenous antigens 28. CBP-12-gp100 substantially inhibited B16 tumor growth (Figure 3B) and increased the percentage of tumor-infiltrating CD8^+^ T cells (Figure 3C and S16A). CBP-12-gp100 immunization also induced potent antitumor immune responses against the melanoma. By contrast, administration of the gp100_25-33_ antigenic peptide alone failed to induce effective antitumor responses (Figure 3D, E and S16B, C). These results indicated that CBP-12 conjugated to an endogenous antigen, in the absence of an adjuvant, successfully induced an antigen-specific CD8^+^ T cell immune response in the anti-PD-1-resistant tumor B16 model.

### CBP-12 stimulates Clec9a^+^ DCs to produce IL-21, which enhances the function of CD8^+^ T cells

We investigated the mechanism involved in the antitumor immune responses elicited by the CBP-12 peptide targeting Clec9a, in the absence of an adjuvant. We found that the mRNA expression level of IL-21 was significantly upregulated in mice treated with CBP-12-gp100, but there was no change in the putative inflammatory cytokine IL-12 (Figure S17), which is induced by many adjuvants 29, 30. The DC maturation markers CD80 and CD86 also showed no significant changes following treatment with the CBP-12 conjugated peptide vaccine (Figure S18).

**Figure 4:**
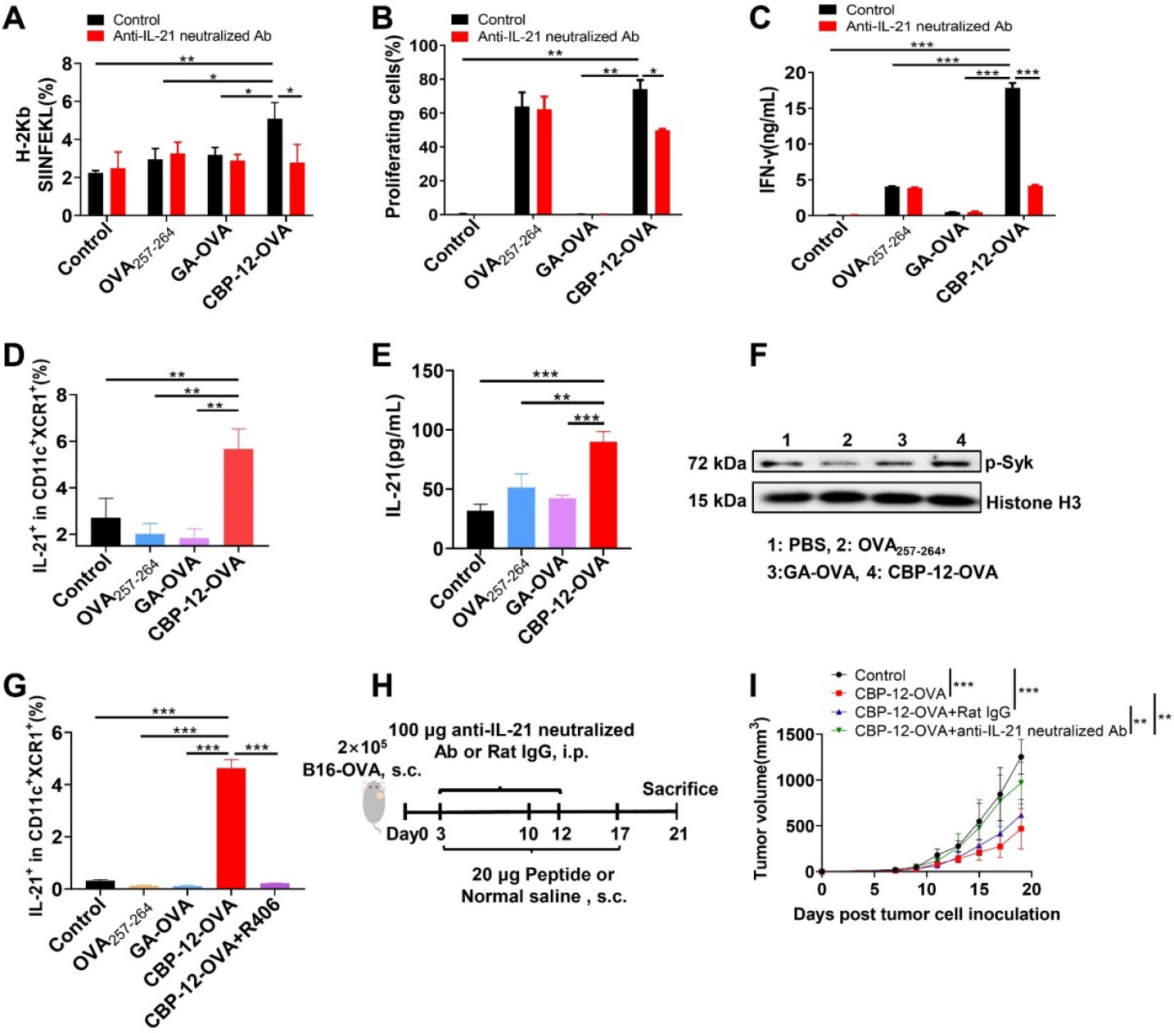
** The CBP-12 peptide vaccine stimulates cDC1s to secrete IL-21.** PBS was used as the negative control. (A) Neutralization of IL-21 significantly decreased the ability of DCs to process the antigen. FL-DCs were incubated with 6 µM of the indicated peptides with or without 5 µg/mL of anti-IL-21 neutralizing antibody for 2 h at 37 °C. Cells were used for OVA_257-264_/H-2K^b^ complex analysis. (B, C) T cell proliferation and IFN-γ secretion were inhibited by neutralizing IL-21. The peptide-loaded FL-DCs were incubated with OT-1 CD8^+^ T cells for 72 h in the presence or absence of 5 µg/mL of anti-IL-21 neutralizing antibody. The cells and supernatants were used to assess T cell proliferation and IFN-γ secretion, respectively. (D) CBP-12-OVA stimulated cDC1s (CD11c^+^XCR1^+^) to secrete IL-21* in vitro*. FL-DCs were incubated with 100 µg/mL of the indicated peptides for 6 h at 37 °C. The frequency of IL-21-positive cells was determined by ICS assay. (E) CBP-12-OVA stimulate cDC1s to secrete IL-21. cDC1s were isolated from FL-DCs and incubated with 100 µg/mL of the indicated peptides for 48 h at 37 °C. The concentration of IL-21 in the supernatant was determined by an ELISA. (F) The level of phosphorylated Syk in FL-DCs was enhanced by CBP-12-OVA. FL-DCs were incubated with 100 µg/mL of OVA_257-264_, GA-OVA, or CBP-12-OVA with 2 mM sodium orthovanadate for 30 min at 37 °C. The level of phosphorylated Syk in FL-DCs was determined by WB. (G) The induction of IL-21 secretion by CBP-12-OVA was inhibited by R406, an inhibitor of Syk phosphorylation. FL-DCs were incubated with 2.5 µM R406 for 1 h, and then cultured with 100 µg/mL of the indicated peptides for 6 h at 37 °C. IL-21 positive frequency was determined by ICS assay. (H) Schematic of the *in vivo* IL-21 neutralization experiment. Three days after tumor cell inoculation, the mice were vaccinated with 20 µg of the indicated peptides or normal saline (negative control), a total of three times at 1-week intervals. The mice were i.p. injected with 100 µg of anti-IL-21 neutralizing antibody on days 3 and 12. (I) Tumor growth curves (*n* = 5). The tumor growth curves were compared using two-way ANOVA. Data are presented as the mean ± SEM, *P < 0.05, **P < 0.01, ***P < 0.001. Data are representative of three independent experiments.

Expression of the OVA_257-264_/H-2K^b^ complex was significantly reduced after neutralizing IL-21 with a blocking mAb (Figure 4A and S19A). Of note, neutralization of IL-21 also markedly suppressed the proliferation and IFN-γ release of antigen-specific CD8^+^ T cells (Figure 4B, C and S19B). To further determine whether IL-21 was secreted by cDC1s, we performed an ICS assay in which FL-DCs were stimulated with CBP-12-OVA *in vitro*. As shown in Figure 4D and S19C, CBP-12-OVA stimulated the secretion of IL-21 from cDC1s. We also confirmed that the CBP-12 peptide and CBP-12-gp100 stimulated the secretion of IL-21 from cDC1s (data not shown). We also performed ELISA to measure the IL-21 level in the supernatant of purified cDC1s stimulated with the peptides. As expected, the supernatant IL-21 level was significantly increased after stimulating cDC1s with CBP-12-OVA (Figure 4E). These results suggest that CBP-12 activates cDC1s to induce IL-21 secretion, which could be involved in the antitumor immune response.

It was reported that Clec9a triggers downstream signaling pathways via Syk kinase and promotes the cross-presentation of dead cell-associated antigens via the Syk phosphorylation pathway 31, 32. Therefore, we determined the level of phosphorylated Syk in FL-DCs stimulated with CBP-12-OVA. This experiment revealed that the level of phosphorylated Syk was enhanced by CBP-12-OVA (Figure 4F). R406, an inhibitor of Syk phosphorylation, could suppress the secretion of IL-21 from cDC1s (Figure 4G and S19C). To explore whether the antitumor effect of CBP-12-OVA was dependent on IL-21, IL-21 neutralized antibody was administrated *in vivo* (Figure 4H). The results showed that the antitumor effect of CBP-12-OVA was remarkably impaired by anti-IL-21 neutralized antibody (Figure 4I). Taken together, these results indicated that the peptide carrier CBP-12 could stimulate the secretion of IL-21 from cDC1s, thus enhancing the antigen cross-presentation and tumor-killing effects of CD8^+^ T cells.

### The CBP-12 conjugated peptide vaccine enhanced the therapeutic efficacy of radiotherapy and prolonged survival in tumor models

**Figure 5:**
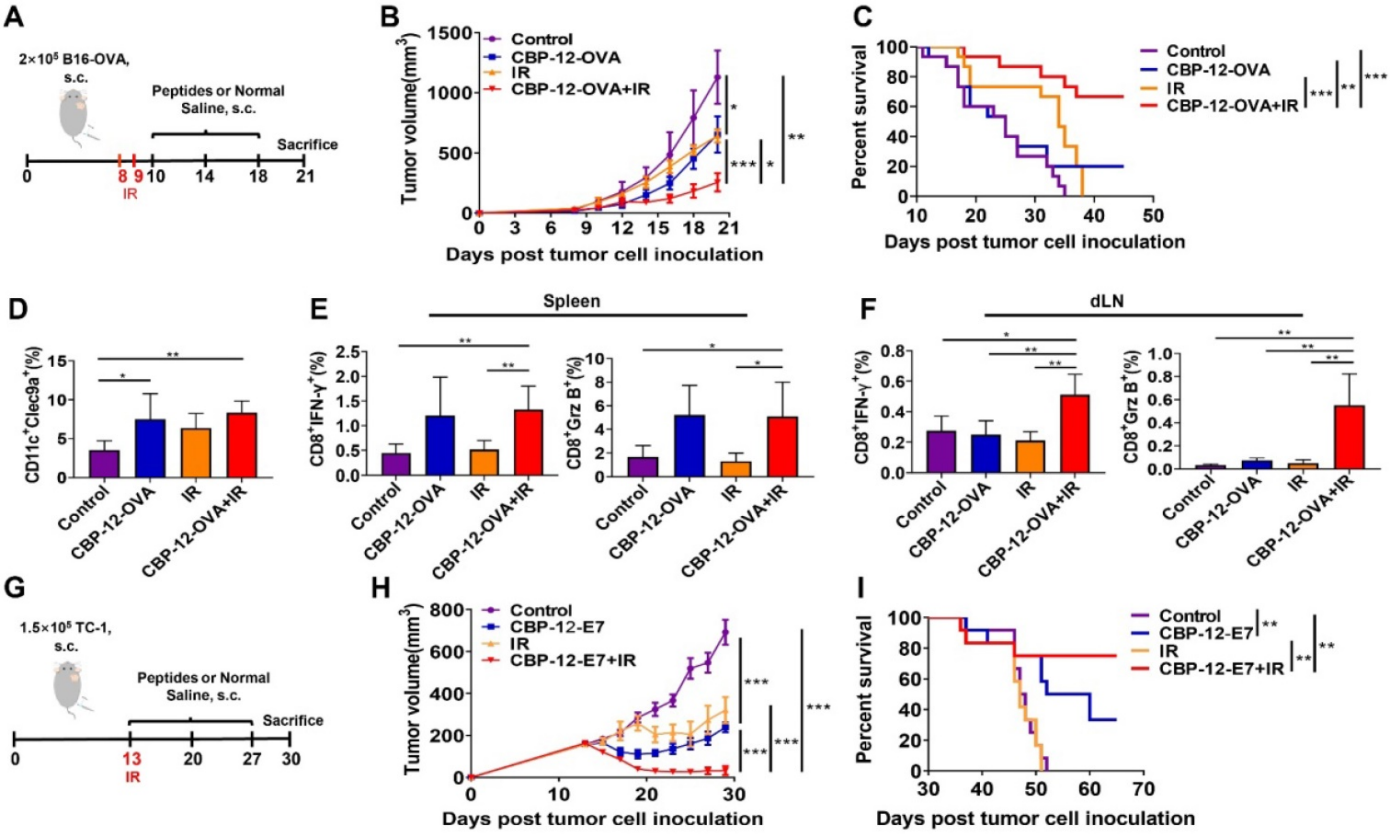
** Administration of the CBP-12 conjugated peptide antigen significantly improved the therapeutic efficacy of radiotherapy.** Normal saline was used as the negative control. (A) Combination therapy schema. When the tumor volume reached approximately 50 mm^3^, the mice underwent local irradiation (IR) with 15 Gy on days 8 and 9. Starting the next day, the mice were vaccinated with 20 µg of CBP-12-OVA or normal saline, a total of three times at 4-day intervals. Three days after the last immunization, the mice were sacrificed. (B) Tumor volumes (*n* = 5). (C) Overall survival analysis of B16-OVA tumor-bearing mice treated as indicated (*n* = 15). (D) Flow cytometry analysis of the proportion of tumor-infiltrating Clec9a^+^ cells (*n* = 4). (E, F) Intracellular IFN-γ and Grz B staining assays of the spleen and dLN (*n* = 4). The cells were re-stimulated with 10 µg/mL of OVA_257-264_ peptide for 6 h *in vitro*, and the antigen-specific T cell responses were analyzed by ICS. (G) TC-1 model schema. When the tumor volume reached approximately 200 mm^3^, the mice underwent local IR at a dose of 20 Gy on day 13. Starting the same day, the mice were vaccinated with 20 µg of CBP-12-OVA or normal saline, a total of three times at 1-week intervals. (H) Inhibition of tumor growth (*n* = 5). (I) Overall survival (*n* = 10). The tumor growth curves were compared using two-way ANOVA. Kaplan-Meier curves and the log-rank test were used to compare overall survival. Data are presented as the mean ± SEM. *P < 0.05, **P < 0.01, ***P < 0.001.

After confirming the antitumor effects of the CBP-12 conjugated peptide vaccines, it was necessary to investigate whether it works synergistically with radiotherapy, which is considered to rebuild the tumor microenvironment and enhance T cell infiltration 33, 34. As shown in Figure 5A, the B16-OVA tumor-bearing mice were vaccinated with the CBP-12 fused antigenic peptide and underwent local irradiation (IR). Strikingly, the tumor volumes were much smaller in the mice treated with CBP-12-OVA combined with IR (Figure 5B), and approximately 60% of the mice in this group survived for at least 45 days (Figure 5C). Although WH-OVA combined with radiotherapy also inhibited tumor growth, the therapeutic effect was weaker than that of CBP-12-OVA (data not shown). In addition, we explored the role of IL-21 in the antitumor responses to combination therapy. The antitumor effect and survival rate following combination therapy was remarkably impaired by administration of an anti-IL-21 neutralizing antibody (data not shown), suggesting that IL-21 plays an important role in the effects of combination radiotherapy. IR alone and combination therapy increased the percentage of Clec9a^+^ DCs in tumor tissues as compared with the normal saline control group (Figure 5D and S20A). We also determined the tumor infiltration of macrophages, CD8^+^ T cells, and CD4^+^ T cells, and found that combination therapy significantly increased the proportions of these cells (Figure S21), indicating that the tumor microenvironment was rebuilt following IR 35. In addition, there were significant increases in the frequencies of IFN-γ- and Grz B-expressing CD8^+^ T cells from the spleen and dLN in the combination therapy group (Figures 5E, F and S20B, C). Furthermore, we examined the curative effects of CBP-12 fused antigenic peptide and radiotherapy in the clinically relevant HPV E6/7 TC-1 tumor model (Figure 5G). In this tumor model, combination therapy significantly inhibited tumor growth (Figure 5H) and more than 70% of mice in the combination therapy group survived for at least 60 days (Figure 5I). Therefore, CBP-12-OVA and CBP-12-E7 combined with radiotherapy inhibited tumor growth and prolonged the survival of tumor-bearing mice. Moreover, the results indicated that the CBP-12 conjugated peptide vaccine could increase the sensitivity of radiotherapy.

## Discussion

Cancer vaccines have demonstrated promising therapeutic effects and several are in late-stage clinical trials, including the WT1-targeted DC vaccine, breast cancer-Her2 pulsed DC vaccine 36, GVAX for patients with hormone-naïve prostate cancer and prostate-specific antigen relapse, PROSTVAC for prostate cancer 8, 34, and the lung cancer-CCL21 DC vaccine 36. However, only Sipuleucel-T has been approved by the US Food and Drug Administration for the treatment of metastatic prostate cancer 34. Most vaccines successfully prevented cancers in mouse models, but the objective clinical responses in advanced cancers remain poor 37. One of the main problems is that the delivery of antigens used in many cancer vaccines is not optimal, and they are unable to maximize immunogenicity 37. Drug delivery barriers are a critical factor that limits vaccine efficacy. Many strategies to overcome these barriers have been attempted, including the use of antibodies, peptides, and drug carrier systems that are capable of delivering the antigens in clinical vaccines 12, 23, 37, 38.

Clec9a is a potential cancer vaccine target that is expressed on a small subset of DCs, which have important immune functions including phagocytosis, oxidative burst, regulation of transcription, cytokine production, cytotoxicity, and antigen cross-presentation 32, 39, 40. Clec9a-targeting antibodies have been used as an antigen carrier, but they cannot induce a CTL response without an adjuvant, resulting in an ineffective response of anti-Clec9a-antigen vaccines *in vivo* 12. Although Toll-like receptor (TLR) agonists are powerful immune stimulators, they could induce an unwanted cytokine release syndrome or autoimmune responses, a major limitation of TLR agonists preventing their clinical application. The delivery of adjuvants and antigens to DCs by targeting DC-restricted receptors was reported to be a feasible tactic that enhances the efficacy and reduces the side effects of adjuvants 12, 21, 22. Adjuvant-free DC vaccines could avoid the side effects associated with the adjuvants, and it was reported that a self-adjuvant nanoemulsion targeting Clec9a with the WH peptide enabled antigen-specific immunotherapy in the absence of an adjuvant 41. Nevertheless, the specificity of the WH peptide was relatively low. Thus, there is a clear need to develop a DC vaccine carrier that effectively delivers the antigen to DC, inducing an antigen-specific CTL response and avoiding the side effects associated with adjuvants.

Here, we report the development of CBP-12, a high-affinity peptide, using an *in silico* optimization method. We found that OVA_257-264_ coupled to CBP-12 (i.e., CBP-12-OVA) greatly enhanced the proliferation of naïve T cells (Figure 1). Thus, CBP-12, as a DC vaccine carrier, overcame the antigen delivery barrier, and efficiently delivered the antigen to Clec9a^+^ DCs. These data are consistent with those of previous studies, which revealed that Clec9a is an endocytic receptor involved in antigen uptake and that, upon antigen binding, the DCs elicited T cell proliferative responses in response to anti-mClec9a-OVA 12-14. We also found that CBP-12-OVA inhibited B16-OVA tumor growth and enhanced antigen-specific CD8^+^ T cells responses, consistent with the conclusion that antigens targeting Clec9a in the presence of an adjuvant promoted tumor immunity 12 (Figure S7). In Clec9a^-/-^ mice, the tumor volume was not significantly different between the CBP-12-OVA and normal saline groups, indicating that CBP-12-OVA specifically targets Clec9a *in vivo* and the antitumor effects are dependent on Clec9a (Figure S7). These results demonstrate that CBP-12, as a DC vaccine carrier, delivers the antigen to Clec9a^+^ DCs and selectively targets Clec9a^+^ DCs *in vivo*. Following this, it was necessary to explore whether CBP-12-OVA can elicit antitumor immune responses in the absence of an adjuvant.

To avoid the unwanted side effects of an adjuvant, we examined whether the targeting Clec9a by CBP-12 can induce a strong CTL response in the absence of an adjuvant. In fact, we found that the adjuvant-free CBP-12-OVA efficiently induced specific CTL responses *in vivo* (Figure S8). These results provide further evidence that CBP-12 can efficiently deliver antigens and shows strong specificity *in vivo*. In the absence of an adjuvant, a low dose of the CBP-12 conjugated peptide vaccine elicited a strong antitumor immune response, indicating that CBP-12 is an ideal vaccine carrier for the delivery of exogenous and endogenous tumor antigens (Figure 2 and 3). In addition, we found that CBP-12 could bind to human Clec9a (data not shown), providing further evidence of its potential clinical use in the future. Earlier studies demonstrated that adjuvants are needed for antigens targeting Clec9a using an antibody to induce antigen-specific antitumor response 12. However, CBP-12 was able to deliver exogenous and endogenous tumor antigens to Clec9a and induced an antigen-specific antitumor response in the absence of an adjuvant, thus eliminating the risk of side effects associated with adjuvants. Adjuvant-free peptide vaccines may greatly accelerate the clinical applications of cancer vaccines. In addition, CBP-12 could be used to deliver various neoantigens for personalized therapies. Moreover, the CBP-12 conjugated peptide vaccine inhibited tumor growth in the anti-PD-1 resistant B16 model. These results demonstrate that the CBP-12-based DC-targeting vaccine is a great platform for treating anti-PD-1 resistant tumor models.

The major role of adjuvant is to stimulate DC maturation and their secretion of cytokine, such as IL-12, which could enhance the function of CD8^+^ T cells. It was reported that Clec9a activation did not affect the DC phenotype or cytokine profile 42. It was also reported that exposure to an adjuvant stimulated CD8^+^ DCs to produce high levels of IL-12 if the CD40 signal was activated 43, 44. When stimulated by an anti-Clec9a delivery system (antigen-Clec9A-TNE), the CD8α^+^ DCs are sufficiently activated by type I IFN, and antigen-specific CD40-CD40L-mediated CD4^+^ T cells help to induce antigen-specific CTL responses in the absence of an adjuvant 41. We found that CBP-12-OVA did not upregulate CD40 expression (data not shown). Unexpectedly, we found that CBP-12 strongly stimulated FL-DCs to produce IL-21, but not IL-12 (Figure S17). IL-21 can be used therapeutically to enhance the killing function and tumor infiltration of CD8^+^ T cells 45. Here, we found no obvious differences in the expression levels of CD80 and CD86 among the groups, suggesting that CBP-12-OVA does not induce DC maturation. In addition, it was reported that IL‑21 has potent inhibitory effects on the activation and maturation of GM‑CSF-induced DCs 46. Thus, we speculate that IL-21 may inhibit the maturation but not the function of FL-DCs. However, when IL-21 was neutralized with a blocking mAb, the expression of OVA_257-264_/H-2K^b^ complex was significantly reduced and the proliferation of antigen-specific CD8^+^ T cells was suppressed, indicating that the Clec9a-targeting peptide mediated antigen cross-presentation was dependent on IL-21 (Figure 4). These findings indicate that a small increase in IL-21 production by cDC1s is vital for cross-presentation of DCs. Indeed, CBP-12-OVA exerted antitumor effects by producing IL-21 *in vivo*. Therefore, CBP-12 could stimulate cDC1s to secret IL-21.

Clec9a activates the downstream signal via Syk kinase, and promotes antigen cross-presentation by phosphorylated Syk 31, 32. The Clec9a cytoplasmic tail contains a hemi-immunoreceptor tyrosine-based activation motif (hemITAM) with a tyrosine at position 7 14, and necrotic cells triggered Clec9a signaling in a Syk- and Tyr7-dependent fashion 31. Clec9a ligation of F-actin caused phosphorylation of tyrosine and activation of Syk family kinases 16, 31, 39. Our results indicate that CBP-12-OVA could stimulate FL-DCs to produce IL-21 by increasing Syk phosphorylation, which was blocked by the Syk inhibitor R406 (Figure 4). This CBP-12 conjugated peptide vaccine stimulated Clec9a^+^ DCs to activate Syk and generate IL-21, which could enhance antigen cross-presentation and the function of CD8^+^ T cells.

Radiotherapy is one of the main conventional cancer treatments that directly causes tumor cell death and augments tumor-specific immunity 47. Patients with advanced melanoma hardly benefit from conventional cancer therapy because melanoma is not sensitive to radiotherapy 7. In this study, we showed that radiotherapy increased the proportion of Clec9a^+^ DCs within the tumor and enhanced the therapeutic effects of the CBP-12 conjugated peptide vaccine (Figure 5). An increase in tumor-infiltrating Clec9a^+^ DCs was important for enhancing the immune response in the tumor microenvironment (Figure S21). These results indicate that the combination therapy exerts its antitumor effect mainly by increasing the percentage of tumor-infiltrating T lymphocytes and inducing antigen-specific T cell responses. Of particular interest, the CBP-12 conjugated peptide vaccine combined with radiotherapy inhibited tumor growth and prolonged survival, which may support the combination of a vaccine and radiotherapy as a new treatment strategy for some advanced cancers.

## Conclusions

**Figure 6:**
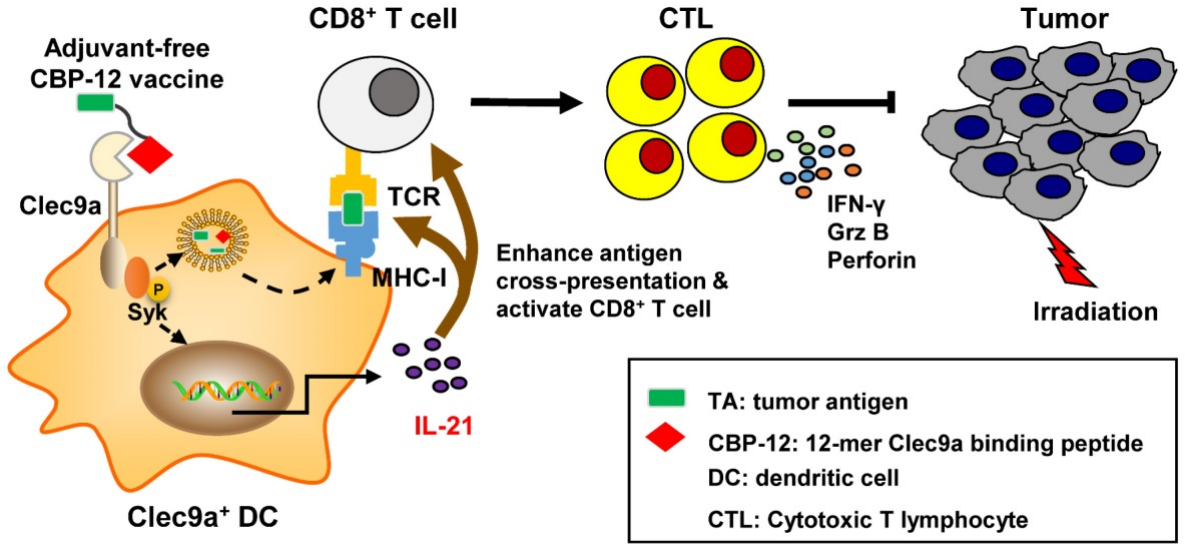
Proposed model by which the adjuvant-free CBP-12 conjugated peptide vaccine elicits an IL-21-dependent antitumor response by targeting Clec9a on DCs.

As summarized in Figure 6, the CBP-12 conjugated peptide vaccine binds to Clec9a on DCs to activate Syk and is internalized via endocytosis. The cross-presentation of the exogenous antigen delivered by the peptide vaccine was mediated and enhanced by Clec9a. The CBP-12 conjugated peptide vaccine stimulated Clec9a^+^ DCs to secrete IL-21, which enhanced antigen cross-presentation and CD8^+^ T cell function. Finally, the CBP-12 conjugated peptide vaccine activated extensive CD8^+^ T cells and induced therapeutic antitumor effects synergistically with radiotherapy. Because this peptide did not induce other inflammatory cytokines, such as IL-12, CBP-12 could serve as an effective carrier to deliver antigens to DCs via Clec9a and simultaneously trigger IL-21 release, avoiding the need for an adjuvant. The CBP-12 conjugated peptide vaccine also elicited therapeutic effects in an anti-PD-1-resistant tumor model.

## Supplementary Material

Supplementary figures and tables.

## Abbreviations

DC: dendritic cell
CBP-12: 12-mer Clec9a binding peptide
ICIs: immune checkpoint inhibitors
CTL: specific cytotoxic T lymphocyte
TAAs: tumor associated antigens
pDC: plasmacytoid DC
F-actin: actin filament
IFN: interferon
HPV: human papilloma virus
BM: bone marrow
RP-HPLC: reverse phase-high performance liquid chromatography
MS: mass spectrometry
SA: streptavidin
MST: microscale thermophoresis
WT: wild type
s.c.: subcutaneous
i.p.: intraperitoneally
dLN: draining lymph node
ICS: intracellular cytokine staining
IR: irradiation
TLR: Toll-like receptor
