# Supplementary material for: Adjuvant-free peptide vaccine targeting Clec9a on dendritic cells can induce robust antitumor immune response through Syk/IL-21 axis
Source: Theranostics. 2021 May 24;11(15):7308–21. doi: 10.7150/thno.56406 (PMC8210616; doi:10.7150/thno.56406)
Supplement: Supplementary file 1 — Supplementary figures and tables. [file thnov11p7308s1.pdf]

Supplementary Materials

Shanshan Gou[1], Shuai Wang[1], Wenwen Liu[1], Guanyu Chen[2], Dongyang Zhang[1], Jiangfeng Du[1], Zhongyi Yan[1], Hongfei Wang[1], Wenjie Zhai[1], Xinghua Sui[2], Yahong Wu[1], Yuanming Qi[1] and Yanfeng Gao[1, 2, *]

[1]School of Life Sciences, Zhengzhou University, Zhengzhou 450001, China.

[2]School of Pharmaceutical Sciences (Shenzhen), Sun Yat-sen University, Shenzhen 518107, China.

Corresponding Author: Prof. Yanfeng Gao

School of Life Sciences, Zhengzhou University, Zhengzhou 450001, China. Or to: School of Pharmaceutical Sciences (Shenzhen), Sun Yat-sen University, Shenzhen 518107, China.

Phone: +86 371 67783235; fax: +86 371 67783235.

E-mail: gaoyf29@mail.sysu.edu.cn.

Mutant peptide design

Based on our previous study, Asp248 and Trp250 were two key residues for Clec9a to bind with WH peptide. In order to improve the binding of WH peptide to Clec9a, a silico residue scanning approach was applied to design high affinity WH mutants. The mutants were predicted by using the module of Residue Scanning mutates function, MOE (Molecular Operating Environments) software (2018 version, Chemical Computing Group Inc, Montreal, Quebec, Canada). Two hundred and twenty peptides were obtained, among which 15 peptides were selected and synthesized for experimental identification.

Phagocytosis assay

For phagocytosis *in vivo*, 6-week-old C57BL/6 mice were s.c. vaccinated with 100 μg biotinylated peptide. After 15 min of immunization, mice were sacrificed. Splenocytes were stained with anti-mouse CD45-FITC (30-F11), anti-mouse CD11c-PerCP-efluor 450 (N418), anti-mouse XCR1-BV650 (ZET) and CD11b-APC (M1/70). Then cells were fixed, permeabilized and further stained with SA-PE for CBP-12 targeting analysis *in vivo*.

For phagocytosis *in vitro*, FL-DCs were incubated with biotinylated GA peptide and CBP-12 for 30 min on ice, then cells were washed three times and culture for 2 h at 37 °C. FL-DCs were stained with anti-mouse CD45-FITC (30-F11), anti-mouse CD11c-PerCP-efluor 450 (N418), anti-mouse XCR1-BV650 (ZET) and CD11b-APC (M1/70) to mark cDC1s and cDC2s. Then FL-DCs were fixed, permeabilized and

further stained with SA-PE. CBP-12 uptake by cDC1s and cDC2s were tested with Flow cytometer.

In vitro enzyme degradation of peptide

Peripheral blood of mice was stand at room temperature for 1 h, centrifuged for 10 min and then the supernatant was acquired. The peptide (1 mM) was added into 10% (V/V) serum saline solution and incubated for 2 h at 37 °C. The samples taken at different time points were added 90% (V/V) acetonitrile-glacial acetic acid mixture and centrifuged for 15 min, then the supernatants were collected. The content of the residual peptide in samples was analyzed at 0 h, 0.25 h, 0.5 h, 1 h and 2 h by RP-HPLC, respectively.

CBP-12-OVA targeting to Clec9a together with adjuvant treated tumor model

C57BL/6 or Clec9a$^{-/-}$ mice were s.c. injected with 2 × 10^5 B16-OVA cells. Three days after the tumor implantation, the mice were treated weekly for total of three times, with either 100 μg peptide or normal saline and 30 μg CpG ODN 1826 as adjuvant. Then the mice were sacrificed after five days of the last injection.

Animal immunization and *ex vitro* CTL priming without adjuvant

For animal immunization and *ex vivo* CTL priming, female C57BL/6 mice of 6-8 weeks were immunized with 20 μg $OVA_{257–264}$, GA-OVA, CBP-12-OVA, or normal saline s.c. injected once a week for total of three times. Five days after the last

injection, mice were sacrificed and *ex vivo* CTL priming assay was performed. Splenocytes were incubated with 10 μg/mL $OVA_{257–264}$ peptide and 1 μL/mL brefeldin A (Becton Dickinson, US) for 6 h to test the ability of $CD8^+$ T cells activation by ICS. Other spleen cells were re-stimulated with 10 μg/mL $OVA_{257–264}$ peptide for five days. The IFN-γ was measured by a Mouse IFN gamma ELISA kit.

Memory T cell analysis

For B16-OVA melanoma model, the mice were sacrificed after the end of CBP-12-OVA peptide vaccine treatment. dLN cells were stained with anti-mouse-CD3-FITC (17A2), anti-mouse-CD8-APC (53-6.7), anti-mouse-CD44-PE (IM7), anti-mouse-CD62L-PerCP-eFluor 710 (DREG-56) or isotype control antibody for memory T cell analysis.

MTT assay

The effect of peptide on the proliferation of B16-OVA cells was determined by a MTT assay. Briefly, cells were seeded into a 96-well plate and treated with various concentrations of peptide for 24, 48 and 72 h at 37°C with 5% CO_2. Cell viability was detected using 20 μL/well of 5 mg/mL MTT reagents (Sigma, St. Louis, MO, USA). MTT reduction was determined by a SpectraMax iD5 (San Francisco, CA, USA).

Tumor infiltrating lymphocyte analysis

For B16-OVA melanoma model, tumor cells were stained with

anti-mouse-CD45-FITC (30-F11), anti-mouse-CD3-PerCP-eFlour710 (17A2) and anti-mouse-CD4-PE (53-6.7) or Rat IgG2a-PE kappa Isotype Control (eBR2a) for tumor infiltrating CD4^{+} T cells analysis. Tumor cells were stained with anti-mouse-CD45-FITC (30-F11), anti-mouse-CD3-PerCP-eFlour710 (17A2) and anti-mouse-NK1.1-PE (53-6.7) or Rat IgG2a-PE kappa Isotype Control (eBR2a) for tumor infiltrating NK cells analysis. Cells were stained with anti-mouse-CD45-FITC (30-F11), anti-mouse-11b-APC (M1/70) and anti-mouse-Ly6G-Brilliant Violet 605™ (1A8) or Rat IgG2a-Brilliant Violet 605™ kappa Isotype Control (eBR2a) for tumor MDCS cells analysis. Single cell suspension was stained with anti-mouse-CD45-FITC (30-F11), anti-mouse-CD3-PerCP-eFlour710 (17A2), anti-mouse-CD4-APC (GK1.5) and anti-mouse-Tim-1-PE (RMT1-4) or isotype control antibody for tumor Th2 cells analysis. Single cell suspension was stained with surface antibody anti-mouse-CD45-FITC (30-F11), anti-mouse-CD4-APC (GK1.5) and anti-mouse-CD25-PE (PC61.5). Permeability cells were then stained with intracellular anti-mouse/Rat Foxp3 PE-Cyanine7 (FJK-16s) or isotype control antibody for Treg cells analysis.

Endotoxin measurement

The concentration of endotoxin in CBP-12-OVA (10 μg/mL) was tested by an endotoxin detection limulus kit (Xiamen Bioendo Technology Co., Ltd., Xiamen, China). Endotoxin test water was used as negative control and the standard product was used as positive control. RAW264.7 cells (2×10^5) were seeded into a 24-well

plate and treated with 10 μg/mL CBP-12-OVA or 1 μg/mL LPS (Sigma, St. Louis, MO, USA) for 24 h at 37 °C with 5% CO_2. TNF-α and IL-6 of RAW264.7 cells mRNA expression level was detected by qRT-PCR. The IL-6 forward and reverse primers are 5'- CTCTGCAAGAGACTTCCATCCAGT-3' and 5'-GAAGTAGGG AAGGCCGTGG-3', respectively. The TNF-α forward and reverse primers are 5'-AGGGTCTGGGCCATAGAACT-3' and 5'-CCACCACGCTCTTCTG TCTA-3', respectively. The β-actin forward and reverse primers are 5'-GTGGCATCCATGAAACTACAT-3' and 5'-GGCATAGAGGTCTTTACGG-3', respectively.

IL-21 and IL-12 mRNA expression by qRT-PCR

IL-21 and IL-12 mRNA expression was examined by qRT-PCR. The IL-21 forward and reverse primers are 5'-CGTATGAGAAAAGGACACCCAA-3' and 5'-GGGAACGAGAGCCTATGAGT-3', respectively. The IL-12 forward and reverse primers are 5'-TCTTAGCCAGTCCCGAAACC-3' and 5'-TTGGTCCCGTGTGATGTCTTC-3', respectively. The β-actin forward and reverse primers are 5'- GTGGCATCCATGAAACTACAT-3' and 5'-GGCATAGAGGTCTTTACGG-3', respectively.

***In vitro* maturation of DC**

Immature FL-DCs were incubated with 100 μg/mL $OVA_{257–264}$, GA-OVA, CBP-12-OVA or PBS for 24 h at 37 °C with 5% CO_2. FL-DCs were harvested,

washed twice with FACS buffer, and then stained with anti-mouse-CD11c-APC (N418), anti-mouse-CD80-PerCP-eFluor 710 (16-10A1), anti-mouse-CD86-FITC (GL1), and detected by a flow cytometry.

Tumor infiltrating macrophages analysis

After the end of CBP-12-OVA peptide vaccine and radiotherapy combination treatment, the mice were sacrificed. Tumor cells were stained with anti-mouse-CD45-FITC (30-F11), anti-mouse-CD11b-APC (M1/70), anti-mouse-F4/80-PerCP-Cyanine5.5 (BM8) or Rat IgG2a- PerCP-Cyanine5.5 Isotype Control (eBR2a) for macrophages analysis.

Supplementary Figures

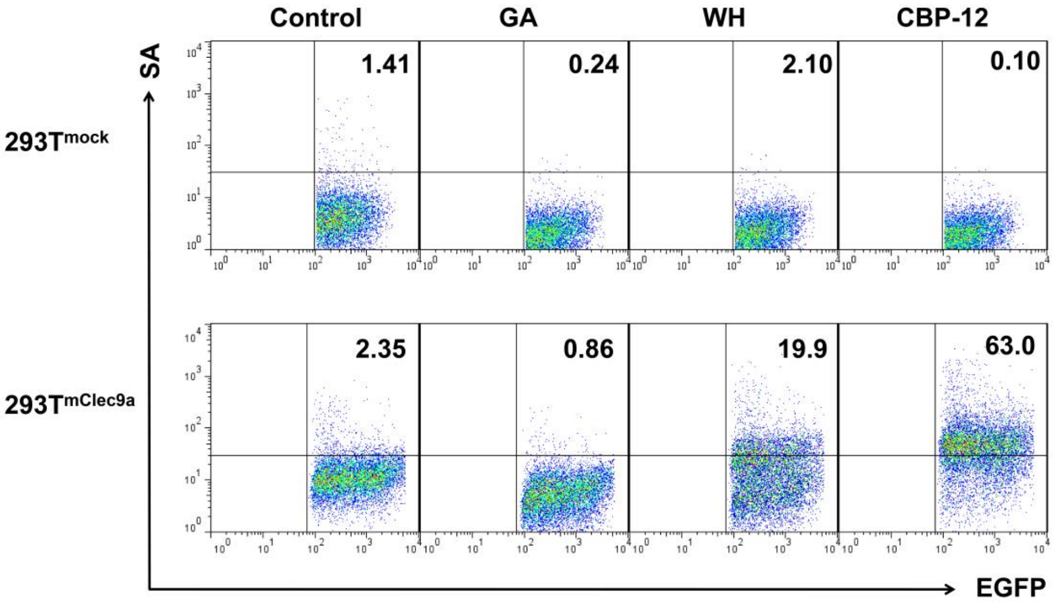

Figure S1. The flow cytometry representative graph of Figure 1A. Binding of CBP-12 and WH peptide to HEK-293T^{mock} and HEK-293T^{mClec9a} cells.

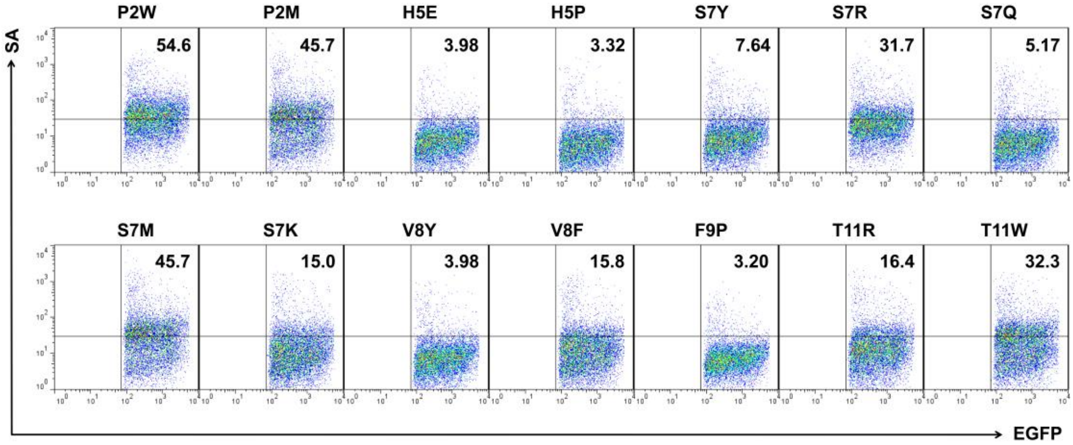

Figure S2. Binding of mutant peptides to HEK-293T^{mClec9a} cells. Each biotinylated peptide (100 μM) was incubated with transfected pIRES2-mClec9a-EGFP cells for 30 min on ice, washed three times with FCS buffer containing 2 mM EDTA, and SA-PE was added to stain the peptide binding to transfected HEK-293T cells.

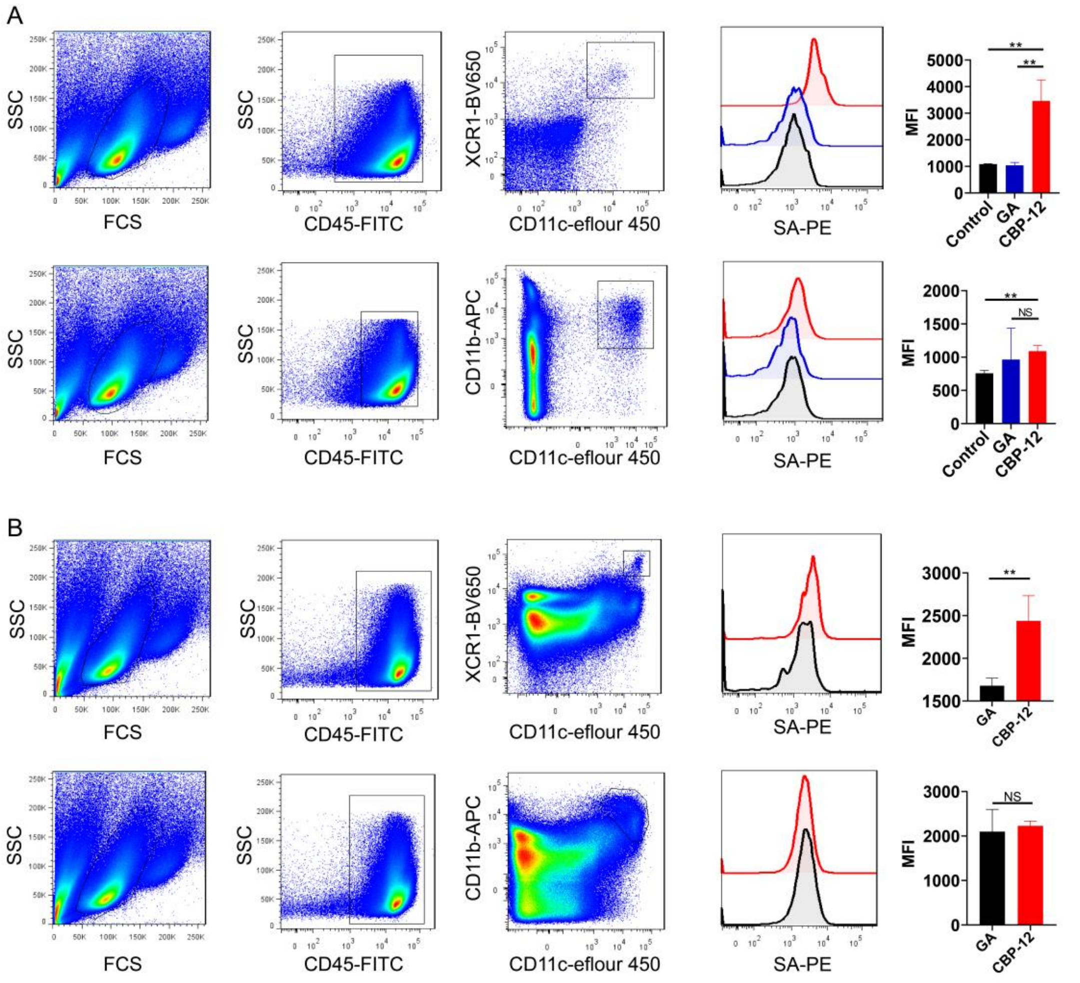

Figure S3. CBP-12 can target to cDC1s *in vitro* and *in vivo.* (A) Biotinylated peptide was uptake by cDC1s and cDC2s of spleen cells *in vitro*. Mouse splenocytes were incubated with 6.25 μM biotinylated GA peptide and CBP-12 for 30 min on ice, then cells were washed three times and cultured for 2 h at 37°C. Spleen cells were gated on cDC1 ($CD45^+CD11c^+XCR1^+$) and cDC2s ($CD45^+CD11c^+CD11b^+$), respectively. Then cells were fixed, permeabilized and further stained with SA-PE for peptide uptake analysis. The histogram and statistics data of cDC1s were cited from Figure 1C. (B) CBP-12 could target to cDC1s but not cDC2s *in vivo*. C57BL/6 mice were vaccinated with 100 μg biotinylated peptide. After 15 min of immunization, mice were sacrificed. Splenocytes were stained with anti-mouse CD45-FITC, anti-mouse CD11c-PerCP-efluor 450, anti-mouse XCR1-BV650 and CD11b-APC. Then cells were fixed, permeabilized and further stained with SA-PE for CBP-12 targeting analysis. Data are presented as the mean ± SEM, NS, no statistical difference, $^{**}P < 0.01$, $n = 3$ per group. Data are representative of three independent experiments.

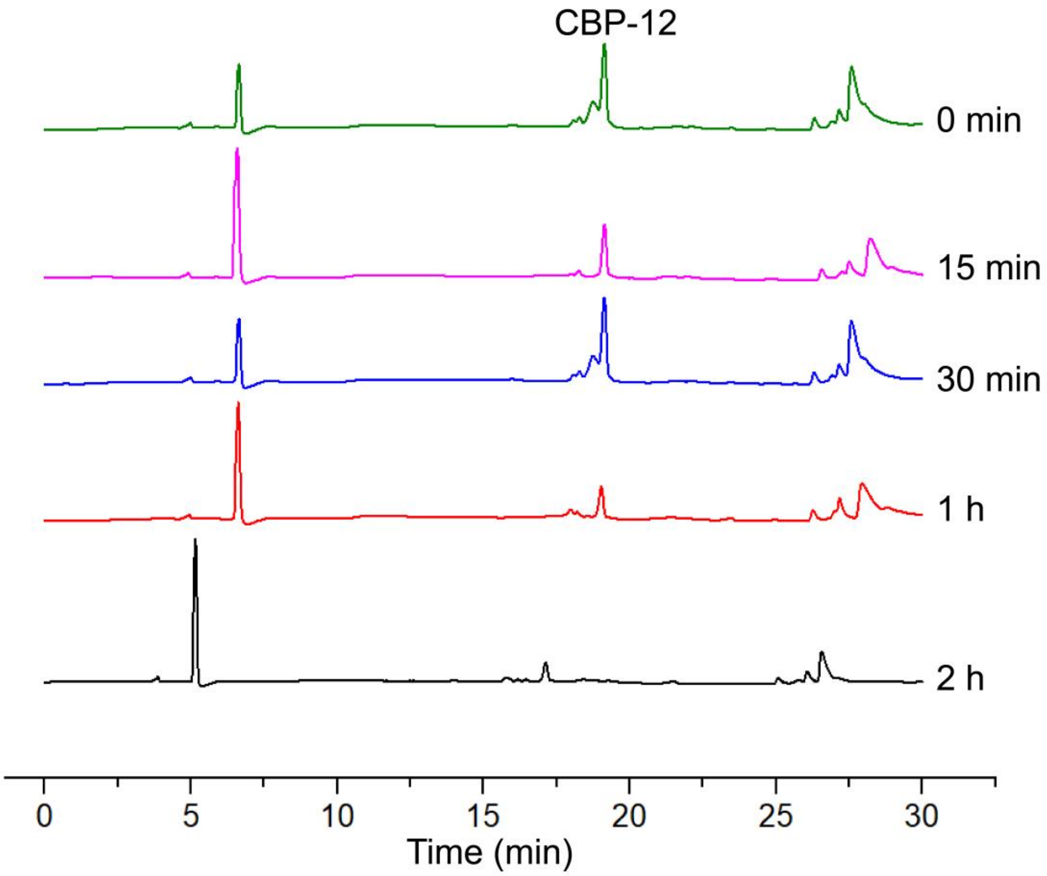

Figure S4. CBP-12 resists protease hydrolysis. The peptide (1 mM) was added into 10% serum saline solution and incubated for 2 h at 37 °C. The content of the residual peptide in samples was analyzed at 0 h, 0.25 h, 0.5 h, 1 h and 2 h by RP-HPLC, respectively.

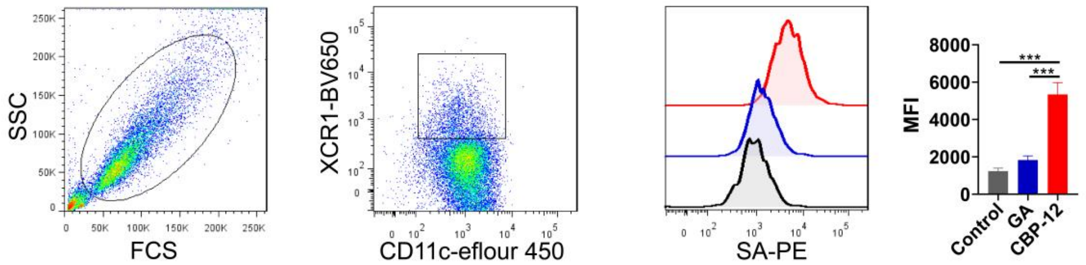

Figure S5. Targeting Clec9a enhances the uptake of CBP-12 by cDC1s *in vitro*. FL-DCs were incubated with 50 μM biotinylated peptide for 30 min on ice, then cells were washed three times and cultured for 2 h at 37 °C. Cells were stained with anti-mouse CD11c-APC, anti-mouse XCR1-BV650 and SA-PE for peptide uptake analysis. Data are presented as the mean ± SEM, ***$P < 0.001$. Data are representative of three independent experiments.

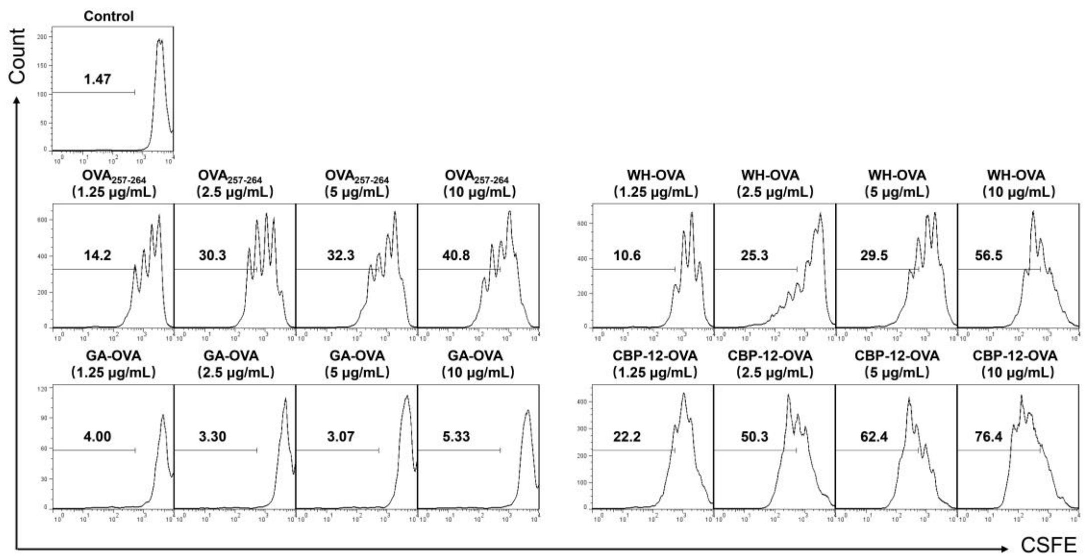

Figure S6. Proliferations of CD8$^+$ T cells. FL-DCs were incubated with different peptides (1.25 to 10 μg/mL) or PBS (negative control) for 1.5 h at 4 °C. CD8$^+$ T cells were isolated from lymph node of OT-1 mice and labeled with 2 μM CFSE. The 1×10^5 CFSE labeled CD8$^+$ T cells were incubated with 1×10^4 peptides loaded FL-DCs for 72 h at 37 °C.

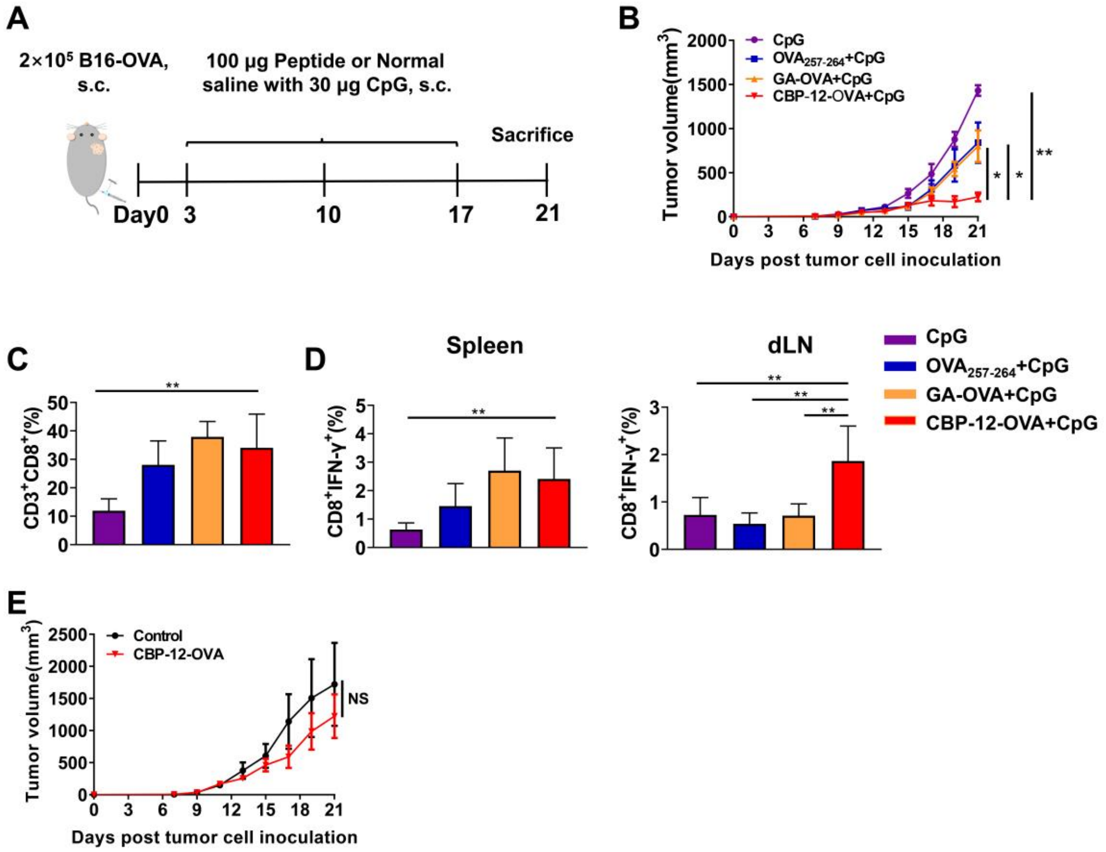

Figure S7. **The antitumor effects of antigen targeting Clec9a in B16-OVA melanoma model.** (A) The scheme of tumor model. B16-OVA melanoma cells (2×10^5) were s.c. injected in right flank of WT or Clec9a$^{-/-}$ mice. On day 3, mice were vaccinated with 100 µg peptides or normal saline (negative control) and 30 µg CpG ODN 1826 weekly for three times. Tumor size was monitored every other day. After five days of last immunization, mice were sacrificed. (B) Tumor volume. (C) Percentage of infiltrating CD8$^+$ T cells in the tumor. (D) Spleen and dLN intracellular CD8$^+$IFN-γ$^+$ T cells frequency. Splenocytes and dLN cells were re-stimulated with 10 µg/mL OVA$_{257–264}$ peptide and brefeldin A for 6 h *in vitro* for ICS analysis. (E) The antitumor effects of CBP-12-OVA peptide vaccine on Clec9a$^{-/-}$ mice. The mice were received treatment similar as described in Figure S7A. The tumor growth curves were compared using two-way ANOVA. Data are presented as the mean ± SEM, NS, no statistical difference, $^*P < 0.05$, $^{**}P < 0.01$. $n = 5$ per group.

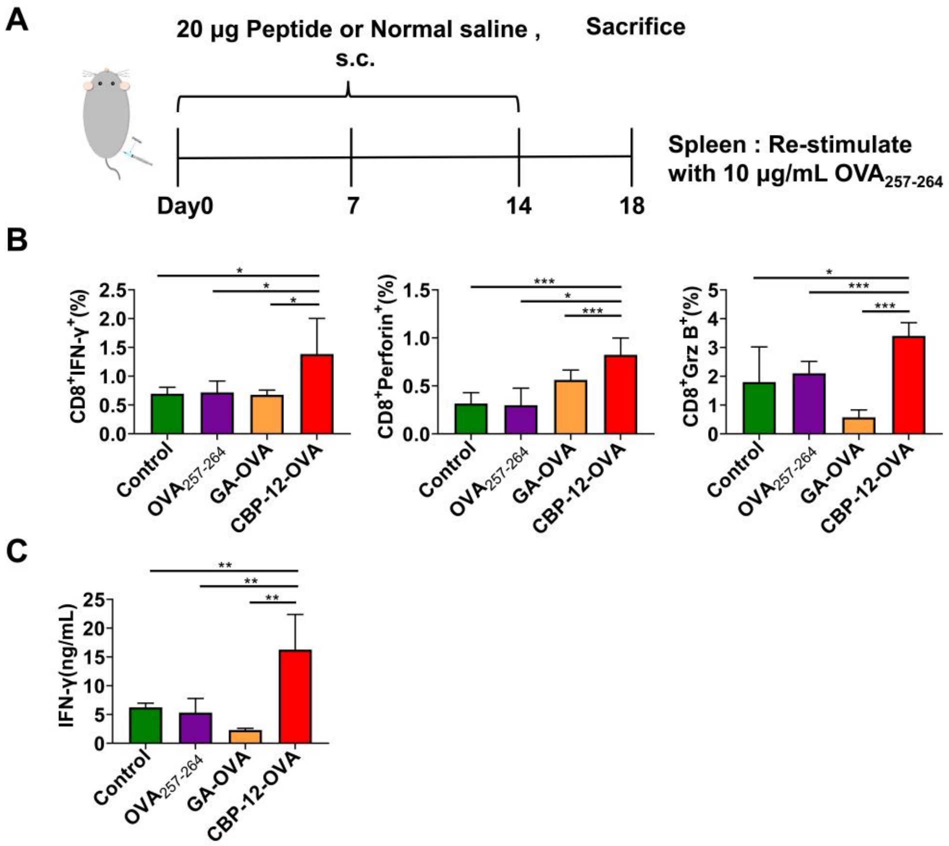

Figure S8. Effects of CBP-12 without adjuvant on inducing of antigen-specific CD8⁺ T cells. Normal saline was used as negative control. (A) Mice were vaccinated with 20 μg different peptides or normal saline weekly for three times. Five days after last immunization, and mouse splenocytes were harvested and re-stimulated with 10 μg/mL $OVA_{257–264}$ peptide *in vitro*. (B) Activated cytotoxic $CD8^+$ T cells were detected by a flow cytometry. (C) ELISA detection for IFN-γ production. Splenocytes were re-stimulated with 10 μg/mL $OVA_{257–264}$ peptide for five days *in vitro,* and IFN-γ from supernatants was measured with ELISA assay. Data are presented as the mean ± SEM, $*P < 0.05$, $**P < 0.01$, $***P < 0.001$. $n = 5$ per group.

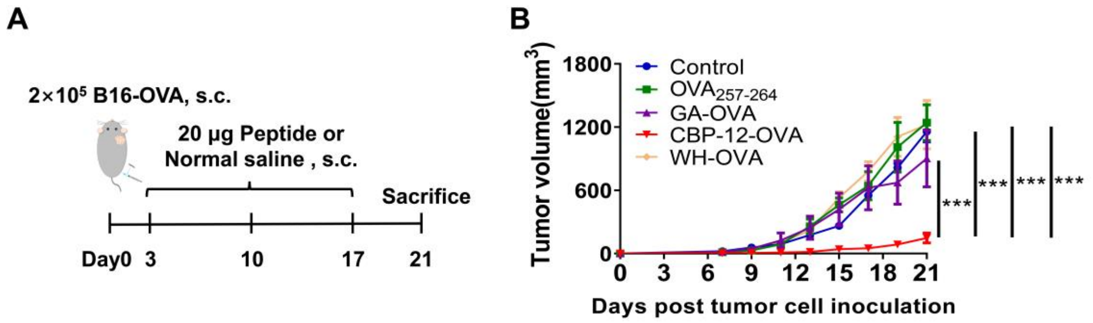

Figure S9. The antitumor effect of WH-OVA in B16-OVA melanoma model. (A) The scheme of tumor model. B16-OVA cells (2×10^5) were s.c. injected in right flank in C57BL/6 mice. On day 3, mice were vaccinated with 20 μg peptide weekly for total of three times. After five days of last immunization, mice were sacrificed. (B) Tumor volume (The tumor growth curves of control group, $OVA_{257\text{–}264}$ group, GA-OVA group and CBP-12-OVA group are cited from Figure 2B). The tumor growth curves were compared using two-way ANOVA. Data are presented as the mean ± SEM, ***$P < 0.001$. $n = 5$ per group.

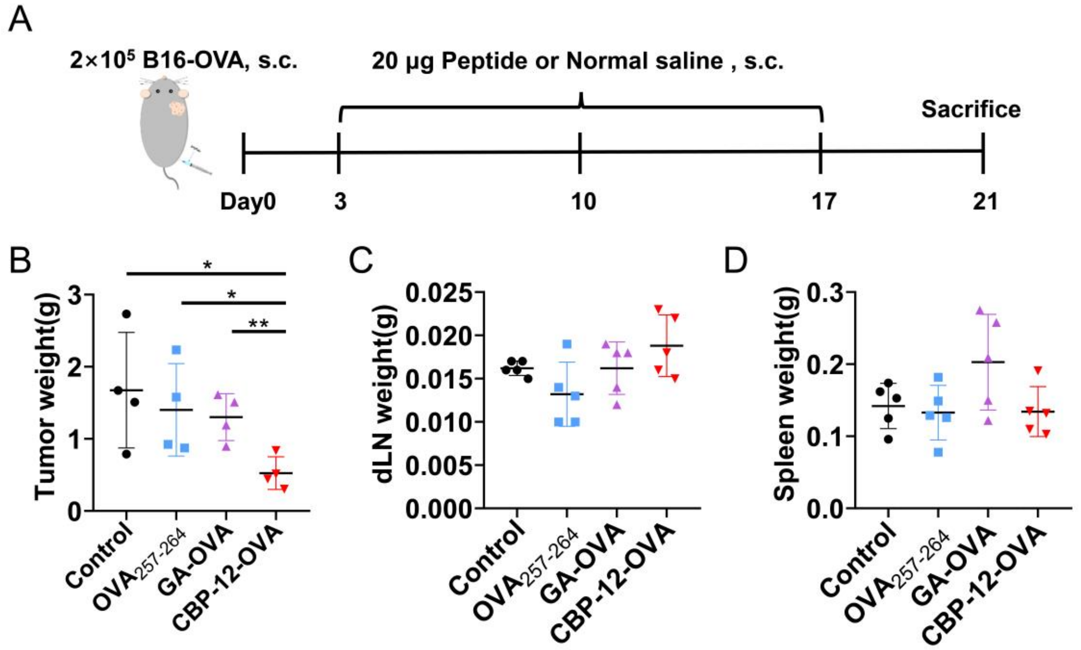

Figure S10. The antitumor effect of CBP-12-OVA in B16-OVA melanoma model. (A) The scheme of tumor model. B16-OVA cells (2×10^5) were s.c. injected in right flank in C57BL/6 mice. On day 3, mice were vaccinated with 20 μg indicated peptides or normal saline (negative control) weekly for total of three times. After five days of last immunization, mice were sacrificed. (B) Tumor weight. (C) dLN weight. (D) Spleen weight. Data are presented as the mean ± SEM, *P

< 0.05, $^{**}P < 0.01$. n = 4-5 per group.

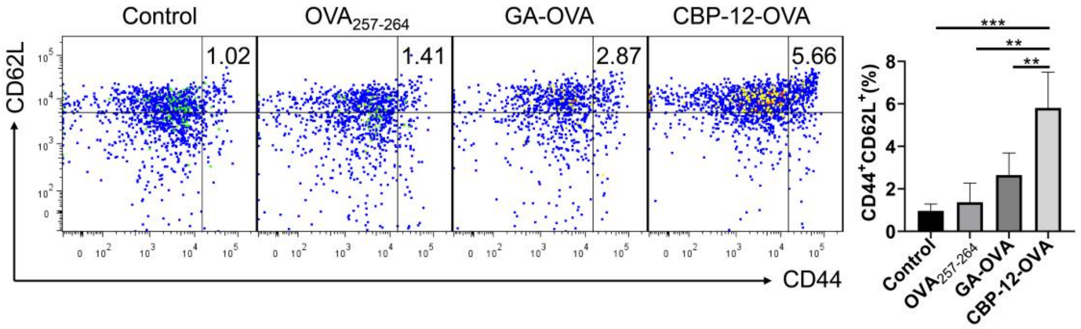

Figure S11. The proportion of memory T cells (CD3$^+$CD8$^+$CD44$^+$CD62L$^+$) in dLN. The mice were received treatment as in Figure 2A. Data are presented as the mean ± SEM, $^{**}P < 0.01$, $^{***}P < 0.001$. n = 5 per group.

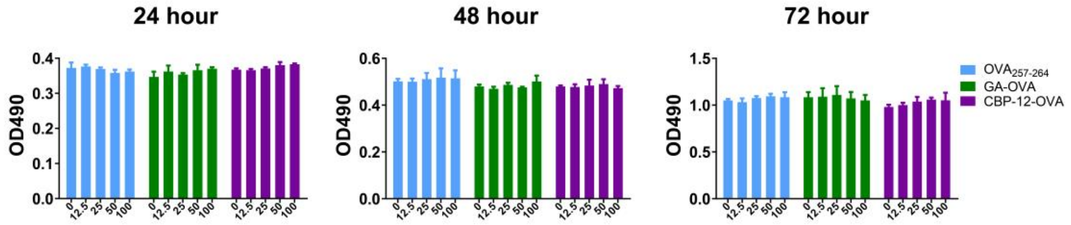

Figure S12. **B16-OVA cells viability after 24, 48 and 72 h treatment with various concentrations of peptide.** Data are presented as the mean ± SEM. Data are representative of three independent experiments.

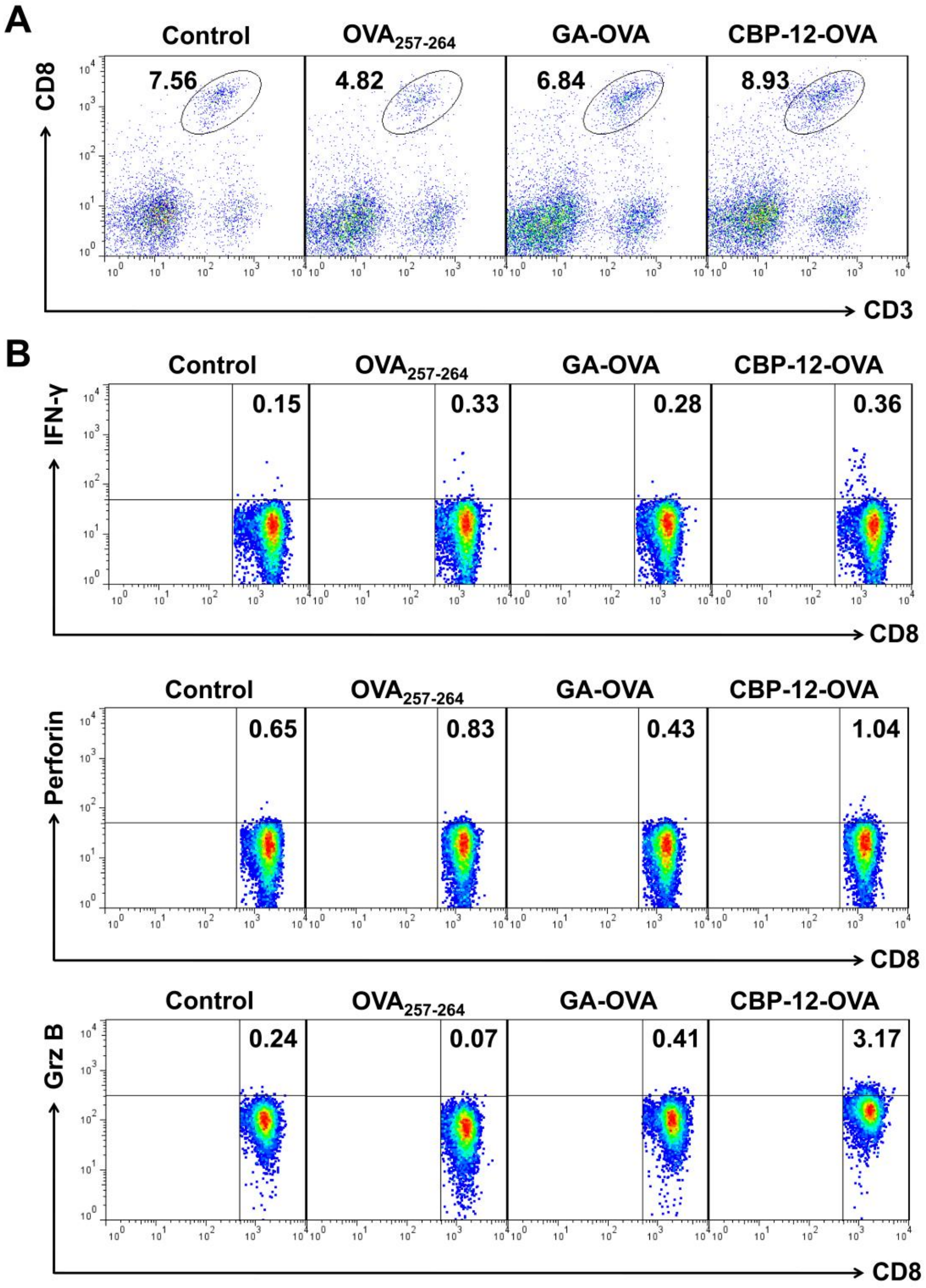

A
Control
OVA257-264
GA-OVA
CBP-12-OVA
CD8
7.56
4.82
6.84
8.93
CD3
B
IFN-γ
0.15
0.33
0.28
0.36
CD8
Perforin
0.65
0.83
0.43
1.04
Grz B
0.24
0.07
0.41
3.17

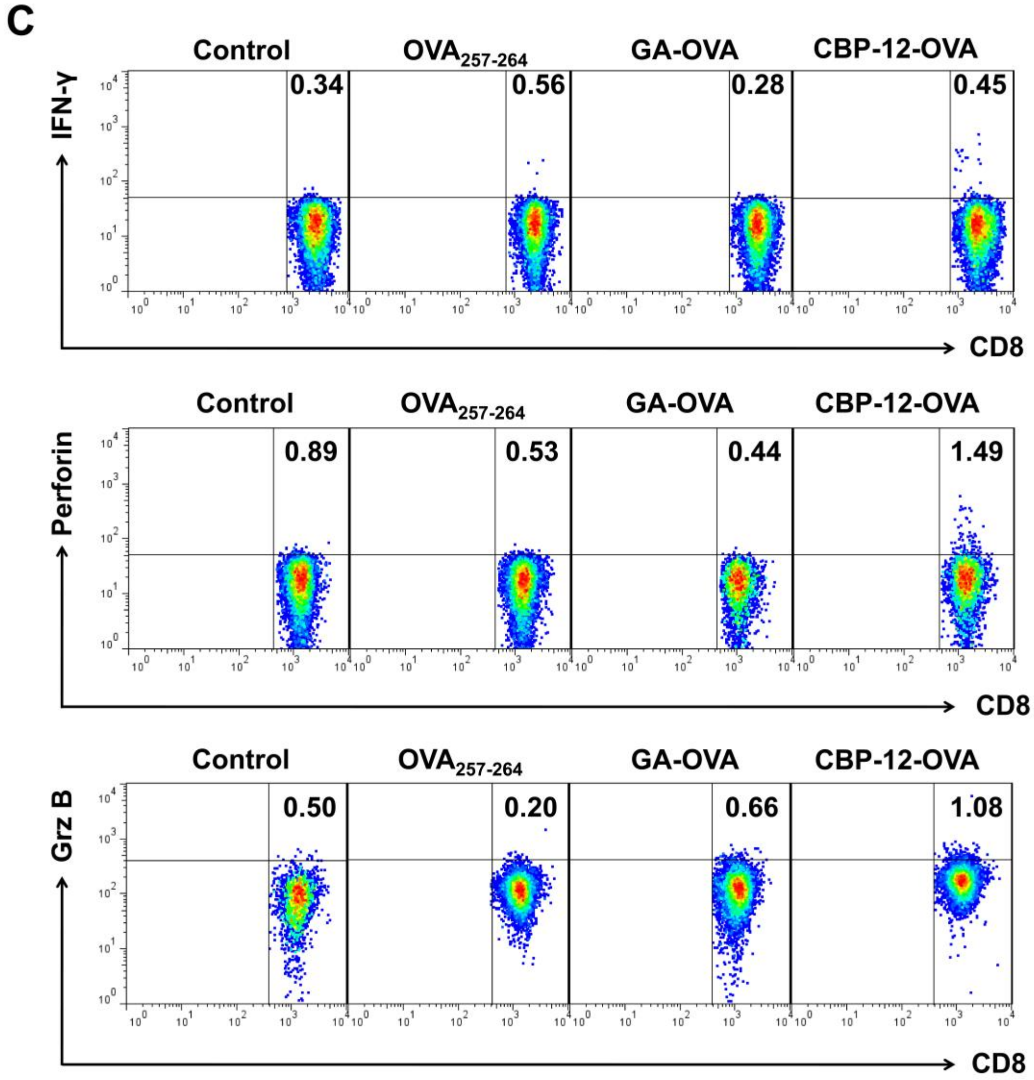

Figure S13. The flow cytometry representative graph of Figure 2. (A) Ratio of infiltrating $CD8^+$ T cells in the tumor. (B, C) The proportion of activated cytotoxic $CD8^+$ T cells in spleen and dLN.

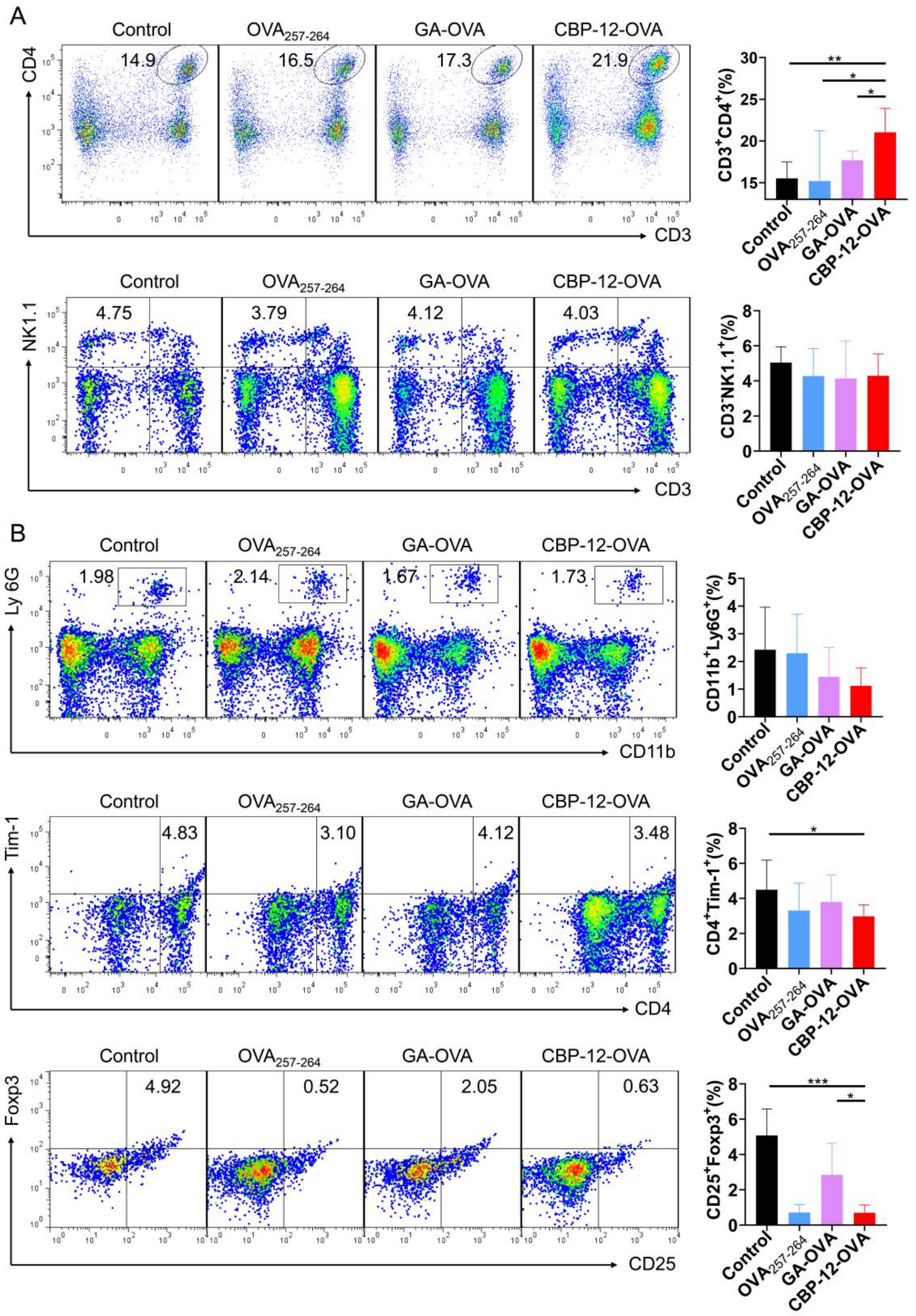

Figure S14. The percentage of tumor infiltrating lymphocytes was detected by a flow cytometry. The mice were received treatment as in Figure 2A. (A) The percentage of $CD4^+$ T cells ($CD45^+CD3^+CD4^+$) and NK cells ($CD45^+CD3^-NK1.1^+$) in tumor. (B) The percentage of MDSC ($CD45^+CD11b^+Ly6G^+$), Th2 ($CD45^+CD3^+CD4^+Tim\text{-}1^+$) and Treg cells ($CD45^+CD4^+CD25^+Foxp3^+$) in tumor. Data are presented as the mean ± SEM, *$P < 0.05$, **$P <$

0.01, ***$P < 0.001$. $n = 5$ per group.

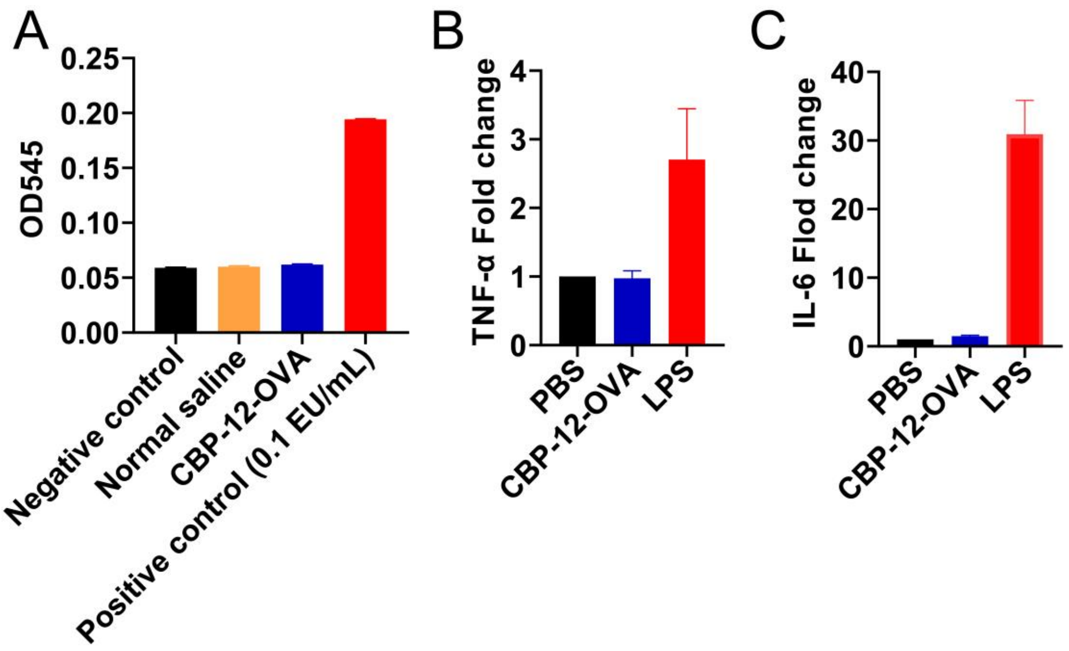

Figure S15. Endotoxin measurements. (A) The endotoxin of CBP-12-OVA (10 μg/mL) was tested by an endotoxin detection limulus kit. (B, C) TNF-α and IL-6 of RAW264.7 cells mRNA expression fold change. 2×10^5 RAW264.7 cells were seeded into a 24-well plate and treated with 10 μg/mL CBP-12-OVA or 1 μg/mL LPS for 24 h at 37 °C with 5% CO_2. Data are presented as the mean ± SEM. Data are representative of three independent experiments.

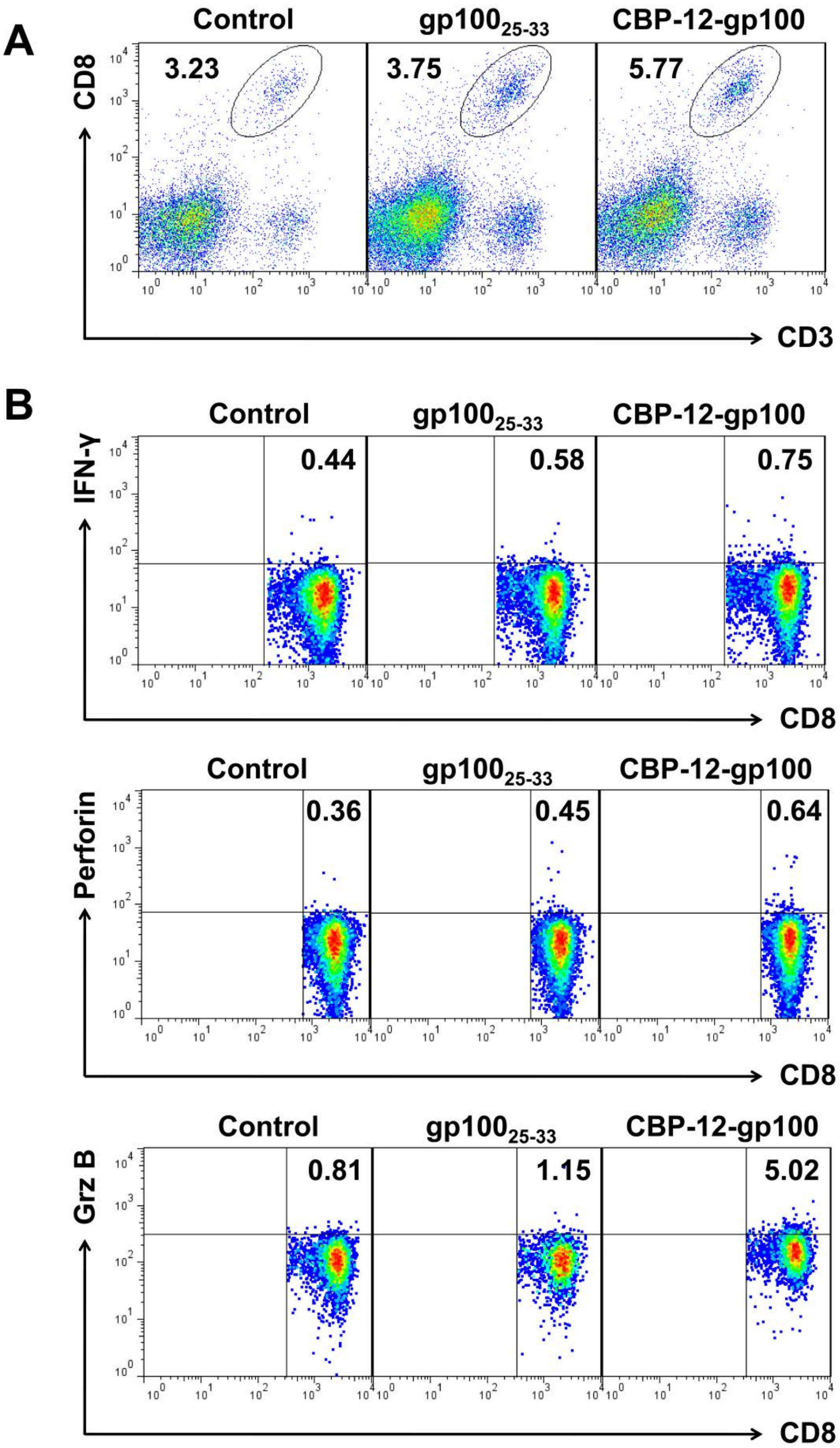
A
Control
gp100 25-33
CBP-12-gp100
3.23
3.75
5.77
CD8
CD3
B
Control
gp100 25-33
CBP-12-gp100
IFN-γ
0.44
0.58
0.75
CD8
Perforin
0.36
0.45
0.64
Grz B
0.81
1.15
5.02

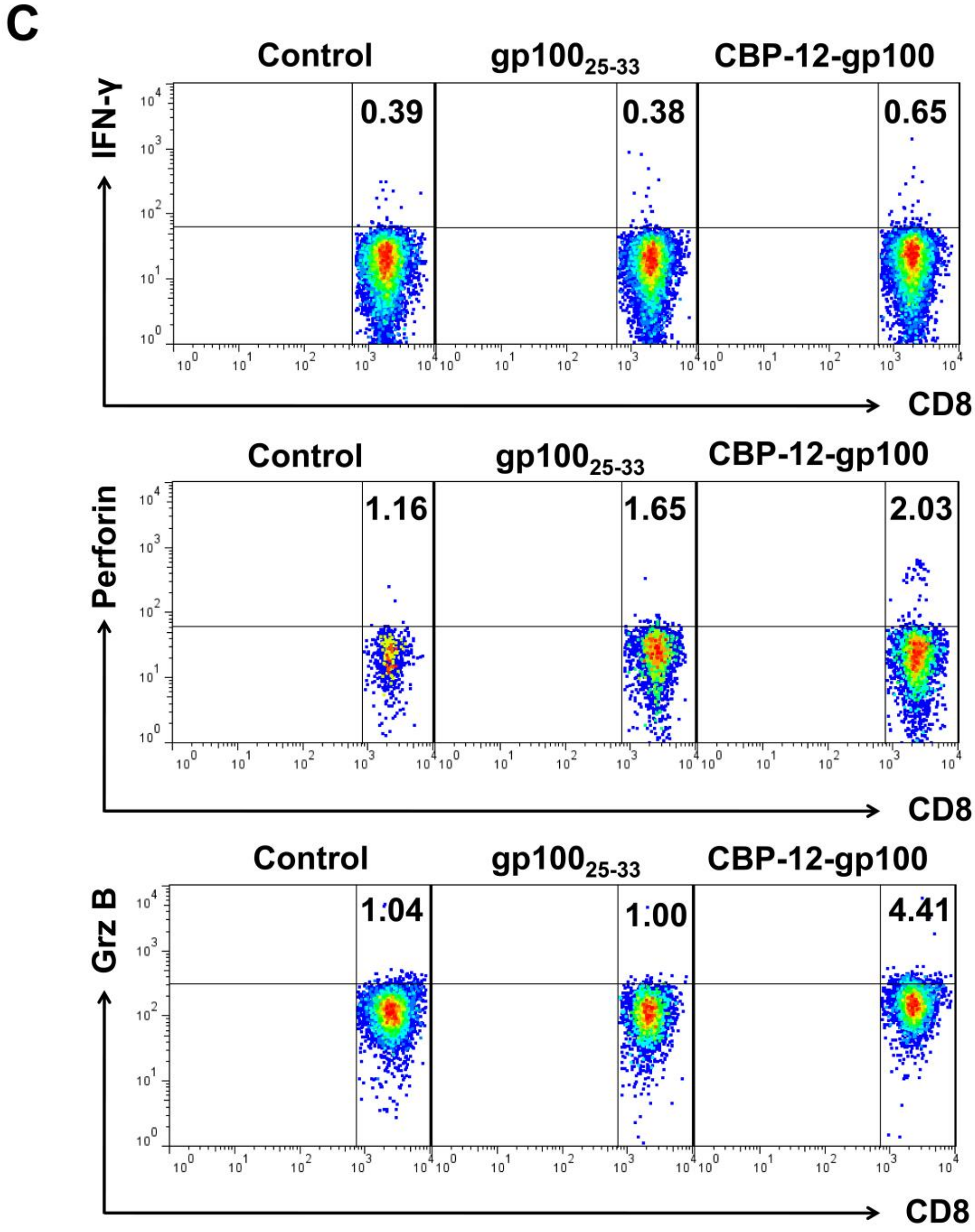

Figure S16. **The flow cytometry representative graph of Figure 3.** (A) Percentage of infiltrating CD8$^+$ T cells in the tumor. (B, C) The proportion of activated cytotoxic CD8$^+$ T cells in spleen and dLN.

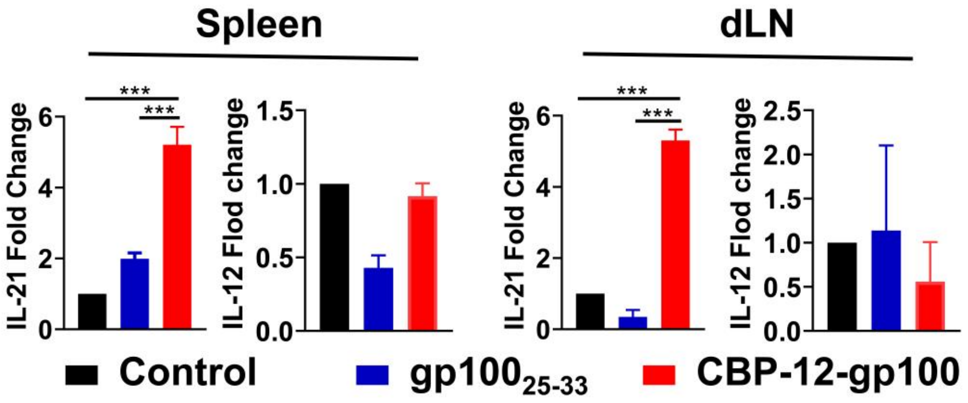

Figure S17. **IL-21 and IL-12 of B16 melanoma cells bearing mice's spleen and dLN mRNA expression fold change.** The mice were received treatment as in Figure 3A. Splenocytes and dLN cells were re-stimulated with 10 μg/mL gp100$_{25–33}$ peptide for five days *in vitro* for IL-21 and IL-12 expression analysis. Data are presented as the mean ± SEM, ****P* < 0.001. Data are representative of three independent experiments.

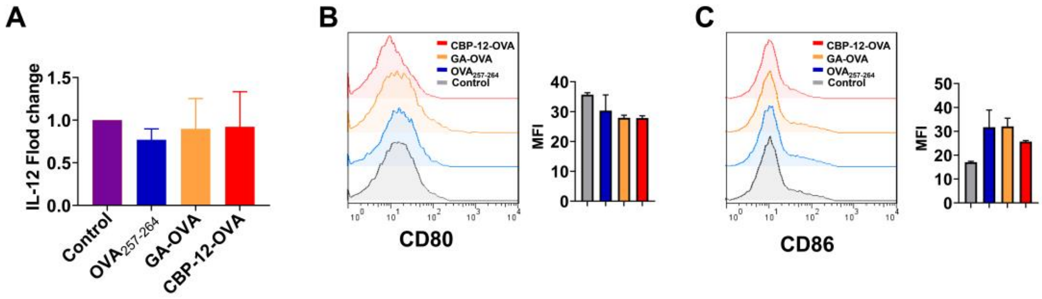

Figure S18. Clec9a triggering does not induce FL-DCs maturation. Immature FL-DCs were cultured with PBS, OVA$_{257–264}$, GA-OVA or CBP-12-OVA for 24 h at 37 °C. (A) IL-12 mRNA expression fold change. (B, C) The expression of co-stimulatory molecules CD80 and CD86 was examined by a flow cytometry, histogram represent statistical result. Data are presented as the mean ± SEM. Data are representative of three independent experiments.

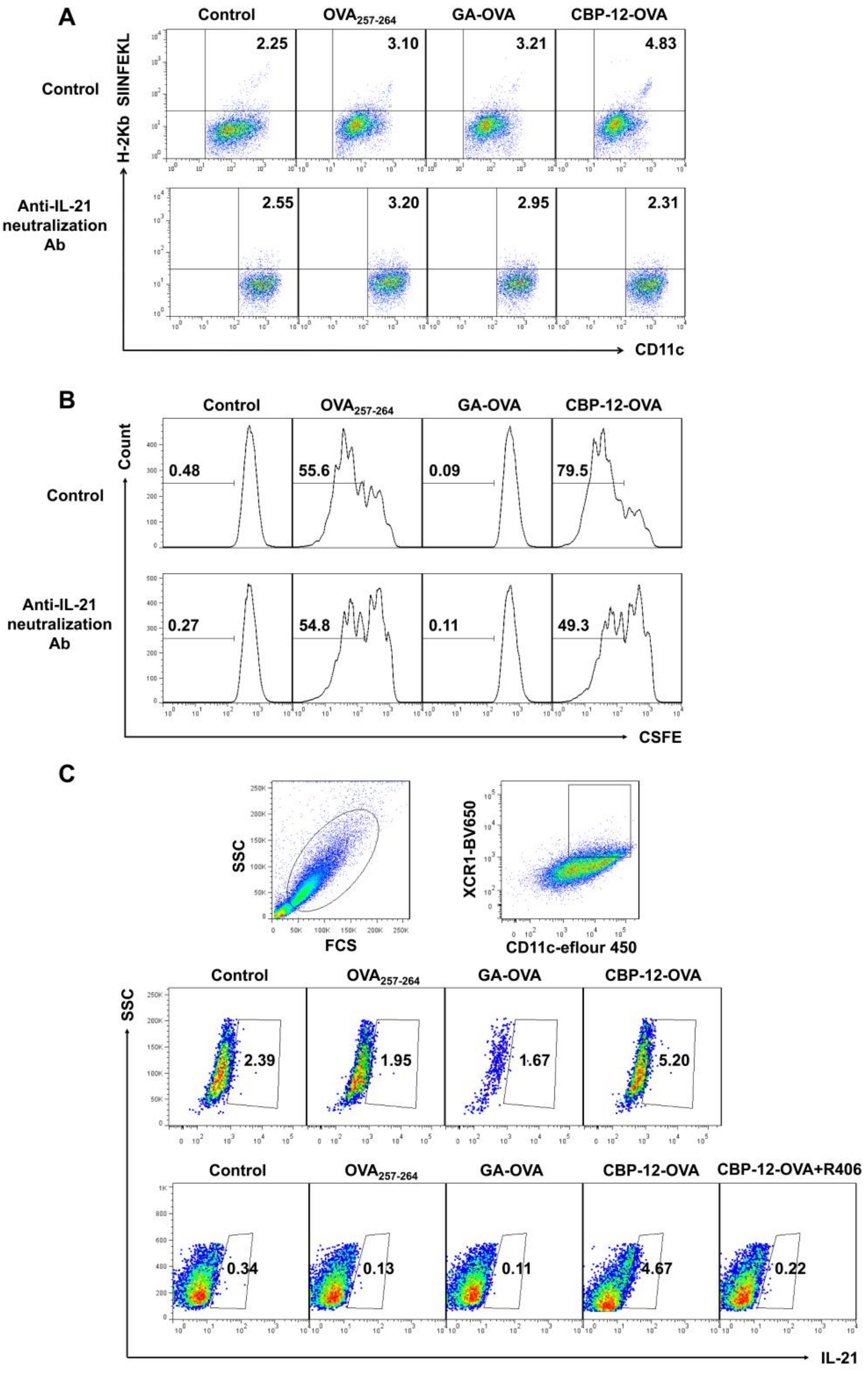

Figure S19. The flow cytometry representative graph of Figure 4. (A) The proportion of $OVA_{257–264}$/H-$2K^b$ complex. (B) The proliferation of $CD8^+$ T cell. (C)The frequency of IL-21 positive cells.

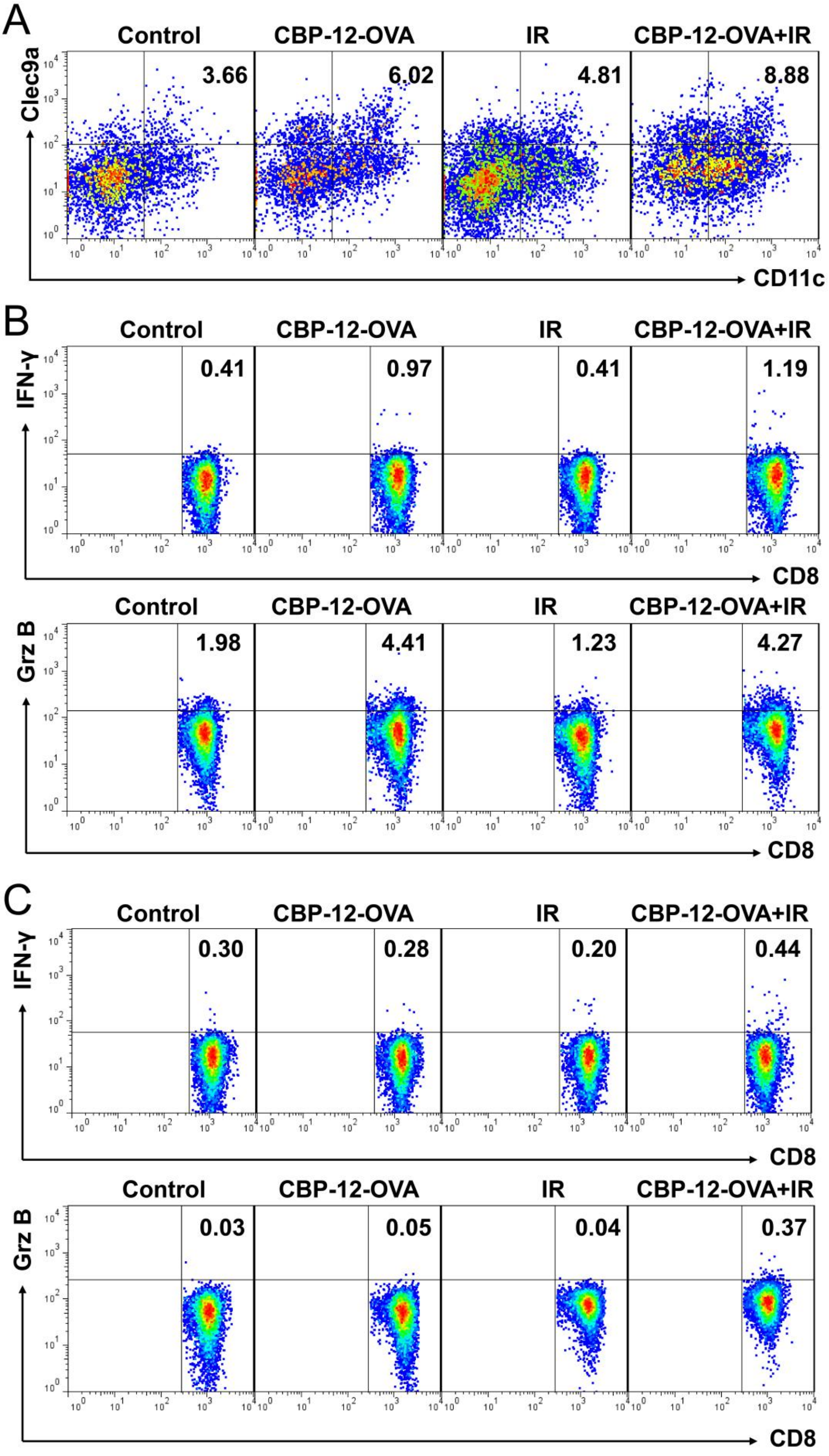
A
Control
CBP-12-OVA
IR
CBP-12-OVA+IR
Clec9a
3.66
6.02
4.81
8.88
CD11c
B
Control
CBP-12-OVA
IR
CBP-12-OVA+IR
IFN-γ
0.41
0.97
0.41
1.19
CD8
Control
CBP-12-OVA
IR
CBP-12-OVA+IR
Grz B
1.98
4.41
1.23
4.27
CD8
C
Control
CBP-12-OVA
IR
CBP-12-OVA+IR
IFN-γ
0.30
0.28
0.20
0.44
CD8
Control
CBP-12-OVA
IR
CBP-12-OVA+IR
Grz B
0.03
0.05
0.04
0.37
CD8

Figure S20. The flow cytometry representative graph of Figure 5. (A) The percentage of infiltrating Clec9a^{+} DCs in the tumor. (B, C) The proportion of activated cytotoxic CD8^{+} T cells in spleen and dLN.

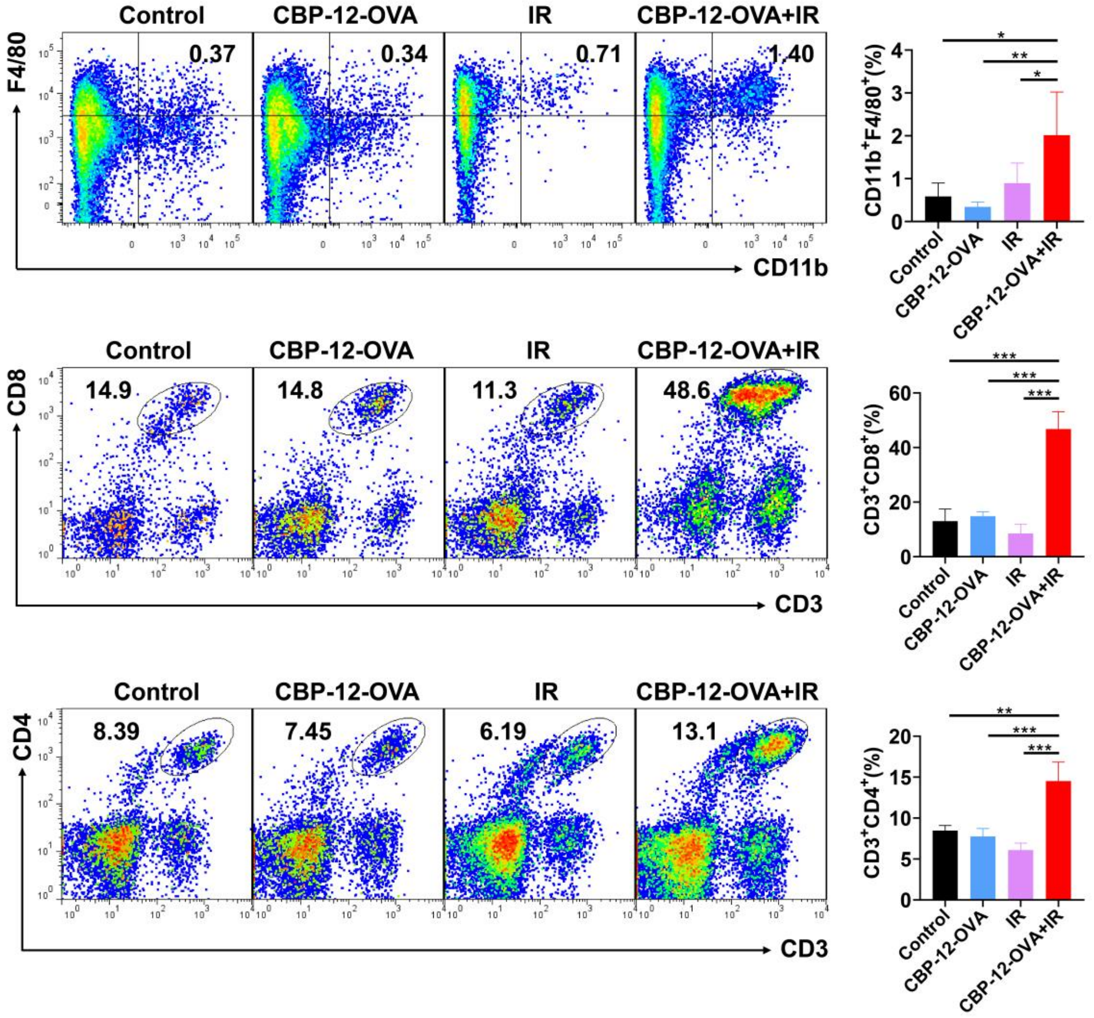

Figure S21. The percentage of tumor infiltrating macrophages, CD8^{+} T cells and CD4^{+} T cells was detected by a flow cytometry. The mice were received treatment as in Figure 5A. Data are presented as the mean ± SEM, *$P < 0.05$, **$P < 0.01$, *** $P < 0.001$. $n = 5$ per group.
